# MicroRNA Mediate Visfatin and Resistin Induction of Oxidative Stress in Human Osteoarthritic Synovial Fibroblasts Via NF-κB Pathway

**DOI:** 10.3390/ijms20205200

**Published:** 2019-10-20

**Authors:** Sara Cheleschi, Ines Gallo, Marcella Barbarino, Stefano Giannotti, Nicola Mondanelli, Antonio Giordano, Sara Tenti, Antonella Fioravanti

**Affiliations:** 1 Department of Medicine, Surgery and Neuroscience, Rheumatology Unit, Azienda Ospedaliera Universitaria senese, Policlinico Le Scotte, 53100 Siena, Italy; ins.gll3@gmail.com (I.G.); sara_tenti@hotmail.it (S.T.); fioravanti7@virgilio.it (A.F.); 2 Sbarro Institute for Cancer Research and Molecular Medicine, Department of Biology, College of Science and Technology, Temple University, Philadelphia, PA 19122, USA; marcella.barbarino@unisi.it (M.B.); giordano@temple.edu (A.G.); 3 Department of Medicine, Surgery and Neurosciences, Section of Orthopedics and Traumatology, University of Siena, Policlinico Le Scotte, 53100 Siena, Italy; stefano.giannotti@unisi.it (S.G.); nicola@nicolamondanelli.it (N.M.)

**Keywords:** microRNA, visfatin, resistin, osteoarthritis, oxidative stress, apoptosis, synovial fibroblasts, synovitis, NF-κB

## Abstract

Synovial membrane inflammation actively participate to structural damage during osteoarthritis (OA). Adipokines, miRNA, and oxidative stress contribute to synovitis and cartilage destruction in OA. We investigated the relationship between visfatin, resistin and miRNA in oxidative stress regulation, in human OA synovial fibroblasts. Cultured cells were treated with visfatin and resistin. After 24 h, we evaluated various pro-inflammatory cytokines, metalloproteinases (*MMPs*), type II collagen (*Col2a1*), *miR-34a, miR-146a, miR-181a*, antioxidant enzymes, and B-cell lymphoma (*BCL)2* by qRT-PCR, apoptosis and mitochondrial superoxide production by cytometry, p50 nuclear factor (NF)-κB by immunofluorescence. Synoviocytes were transfected with miRNA inhibitors and oxidative stress evaluation after adipokines stimulus was performed. The implication of NF-κB pathway was assessed by the use of a NF-κB inhibitor (BAY-11-7082). Visfatin and resistin significantly up-regulated gene expression of interleukin *(IL)-1β*, *IL-6*, *IL-17*, tumor necrosis factor *(TNF)-α,* *MMP-1*, *MMP-13* and reduced *Col2a1*. Furthermore, adipokines induced apoptosis and superoxide production, the transcriptional levels of *BCL2*, superoxide dismutase (*SOD*)*-2*, catalase (*CAT*), nuclear factor erythroid 2 like 2 (*NRF2*), *miR-34a*, *miR-146a,* and *miR-181a*. MiRNA inhibitors counteracted adipokines modulation of oxidative stress. Visfatin and resistin effects were suppressed by BAY-11-7082. Our data suggest that miRNA may represent possible mediators of oxidative stress induced by visfatin and resistin via NF-κB pathway in human OA synoviocytes.

## 1. Introduction

Osteoarthritis (OA) is the most prevalent musculoskeletal disease characterized by a progressive degradation of articular cartilage, osteophyte formation, subchondral sclerosis and synovitis [1,2]. Increasing evidence suggests that synovial membrane inflammation is implicated in the pathophysiology of the disease; prostaglandins, leukotrienes, reactive oxygen species (ROS), cytokines, chemokines and adipokines, produced by inflamed synovium, induced cartilage degradation and further bolster inflammation [3,4,5].

Adipokines, including adiponectin, chemerin, leptin, resistin, and visfatin, are secreted by white adipose tissue and are known to be involved in multiple biological processes, as immunity, inflammation, cartilage and bone metabolism. Much attention has been paid regarding their implication in the pathogenesis of many rheumatic diseases, even OA [6,7,8,9,10]. 

Visfatin has originally identified as an insulin-mimetic factor, with pro-inflammatory and immunomodulating functions [11], while resistin is implicated in obesity-associated insulin resistance and involved in inflammatory response [12].

Visfatin and resistin serum levels and synovial fluid were found to be increased in patients with knee and hand OA [9,13,14,15]; moreover, it has been highlighted the pro-inflammatory effect of these adipokines on the expression of different cytokines and chemokines, as well as their role in mediating the production of matrix degrades enzymes in human OA chondrocytes and synovial fibroblasts [16,17,18,19].

Recent studies demonstrated a complex interaction between adipokines and microRNAs (miRNA) [17,18,20,21]. miRNA are an abundant class of conserved double stranded non-coding RNA molecules of 22–25 nucleotides that are classified as important post-transcriptional regulators of gene expression of target gene messenger RNA [22]. They are implicated in important physiological cellular processes as well as in the pathophysiology of different disorders, including OA [23,24,25,26]. Some miRNA, also known as oxidative stress-responsive factors, can be induced or suppressed by ROS, and their biological function, through regulation of target genes, should be influenced [27]; besides, a specific modulation of oxidative stress balance by specific miRNA has been postulated [28]. 

In the present study, we investigated the complex cross-talk between visfatin, resistin and some miRNA (*miR-34a, miR-146a*, and *miR-181a*) in the regulation of oxidative stress, in human OA synovial fibroblasts.

In particular, we analyzed the effect of visfatin and resistin in gene expression of interleukin (*IL*)*-1β*, *IL-6*, *IL-17A*, tumor necrosis factor (*TNF*)-*α*, metalloproteinases (*MMP*)-*1*, *MMP-13*, collagen type II (*Col2a1*). Furthermore, the apoptotic cells and the transcriptional levels of the anti-apoptotic marker B-cell lymphoma (*BCL) 2*, as well as the production of mitochondrial superoxide anion and the gene levels of antioxidant enzymes [superoxide dismutase (*SOD*)-*2*, catalase (*CAT*)] and nuclear factor erythroid 2 like 2 (*NRF2*) were also investigated. 

To examine the potential role of *miR-34a, miR-146a*, and *miR-181a* as mediators of the visfatin and resistin effects on oxidative stress, we transfected synovial fibroblasts with miRNA specific inhibitors.

Finally, the possible implication of nuclear factor (NF)-κB pathway in adipokines-mediated effects was assessed.

## 2. Results

### 2.1. Cell viability Evaluation in Visfatin and Resistin Treated Cells

Cell viability assay was analyzed by 3-(4,4-dimethylthiazol-2-yl)-2,5-diphenyl-tetrazoliumbromide (MTT) test and the results are represented in Figure S1. A significant reduction of the percentage of survival cells was observed in human OA synovial fibroblasts incubated with visfatin 5 μg/mL and 10 μg/mL (*p* < 0.05) and resistin 50 ng/mL and 100 ng/mL (*p* < 0.05), in comparison to basal condition.

### 2.2. Visfatin and Resistin Promote Inflammation and Regulate Cartilage Turnover

**Figure 1:**
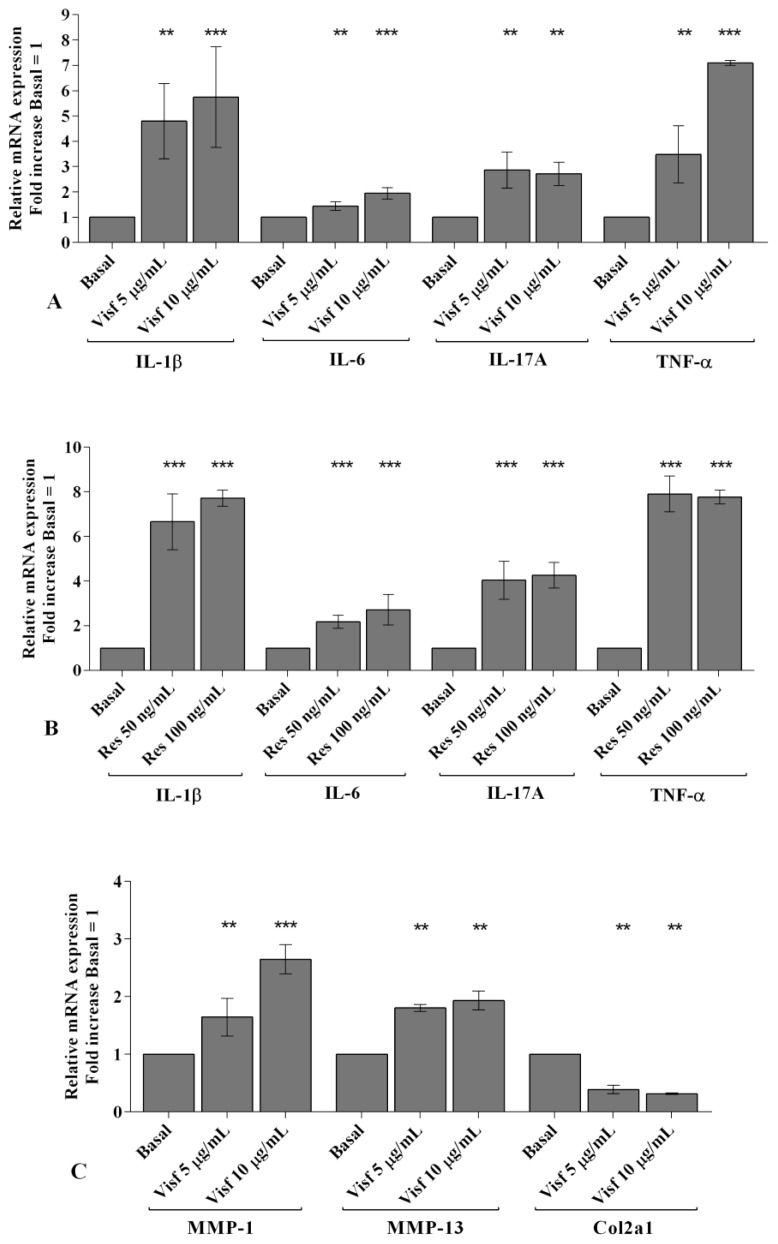

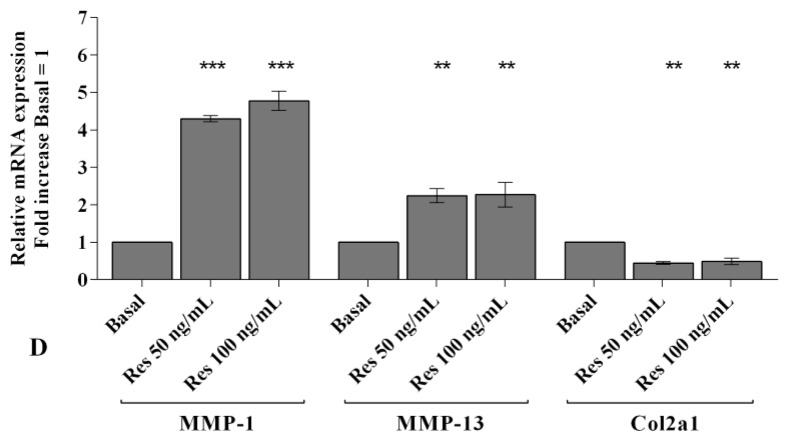
(**A**–**D**) Expression levels of interleukin (*IL*)*-1β*, *IL-6*, *IL-17A*, tumor necrosis factor (*TNF*)-*α*, metalloproteinases (*MMP*)-*1*, *MMP-13,* and collagen type II (*Col2a1*) by real-time PCR. Human osteoarthritic (OA) synovial fibroblasts were evaluated at basal condition and after incubation with visfatin (5 and 10 μg/mL) and resistin (50 and 100 ng/mL) for 24 h. The gene expression was referenced to the ratio of the value of interest and the value of basal condition (basal, cells without treatment), reported equal to 1. Data were expressed as mean ± SD of triplicate values. ** *p* < 0.01, *** *p* < 0.001 versus basal condition. Visf = visfatin, Res = resistin.

The effect of adipokines on gene expression of the main pro-inflammatory mediators IL-1β, IL-6, Il-17A and TNF-α in human OA synovial fibroblasts is reported in Figure 1.

Visfatin, tested at both concentrations, 5 μg/mL and 10 μg/mL, significantly increased the mRNA expression of *IL-1β*, *IL-6*, *IL-17A*, and *TNF-α* (*p* < 0.01, *p* < 0.001) (Figure 1A), in a dose dependent manner. Similarly, resistin 50 and 100 ng/mL induced a significant up-regulation (*p* < 0.001) of gene levels of the studied cytokines compared with the un-stimulated cells (Figure 1B). 

In Figure 1C,D we summarized the regulation of the main extracellular matrix (ECM) degrading enzyme, MMP-1, MMP-13, and of the main component of articular ECM, Col2a1.

In human OA synovial fibroblasts stimulated with visfatin 5 and 10 μg/mL (Figure 1C) and resistin 50 ng/mL and 100 ng/mL (Figure 1D) we showed a significant increase of *MMP-1*, *MMP-13* (*p* < 0.01, *p* < 0.001) and a reduction of *Col2a1* (*p* < 0.01, *p* < 0.001) expression levels, in comparison to basal time.

### 2.3. Adipokines Induce Apoptosis and Regulate BCL2 Expression

**Figure 2:**
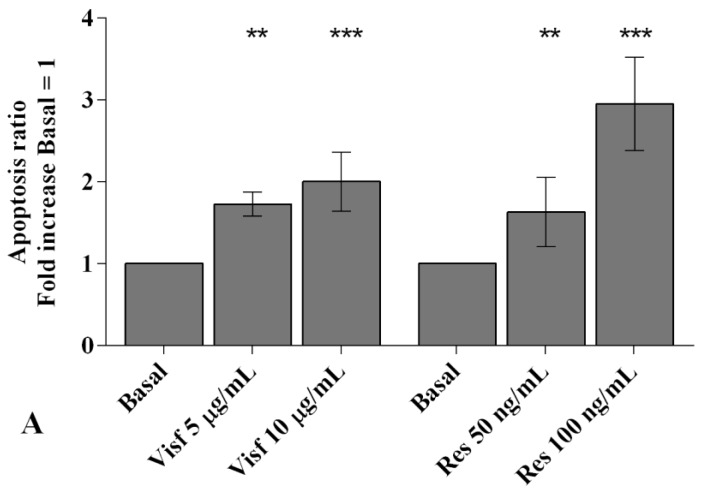

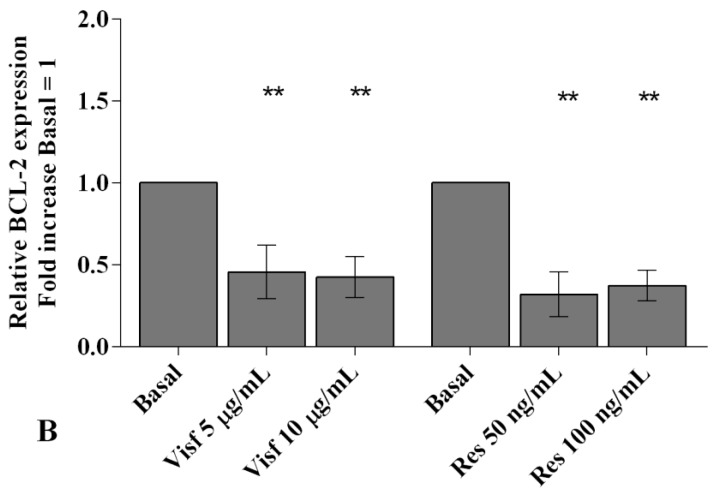
(**A**) Apoptosis detection performed by the analysis at flow cytometry and measured with Annexin Alexa fluor 488 assay. Data were expressed as the percentage of positive cells for Annexin-V and propidium iodide (PI) staining. (**B**) Expression levels of gene B-cell lymphoma (*BCL*)*2* by real-time PCR. Human osteoarthritic (OA) synovial fibroblasts were evaluated at basal condition and after incubation with visfatin (5 and 10 μg/mL) and resistin (50 and 100 ng/mL) for 24 h. The apoptosis ratio and the gene expression were referenced to the ratio of the value of interest and the value of basal condition (basal, cells without treatment), reported equal to 1. Data were expressed as mean ± SD of triplicate values. ** *p* < 0.01, *** *p* < 0.001 versus basal condition. Visf = visfatin, Res = resistin.

Visfatin (5 and 10 μg/mL) and resistin (50 and 100 ng/mL) stimulation induced a significant and dose-dependent increase (*p* < 0.01, *p* < 0.001) of apoptotic OA synovial fibroblasts in comparison to baseline (Figure S2 and Figure 2A).

Real-time PCR analysis underlines a significant reduction of the expression levels of the anti-apoptotic marker *BCL2* (*p* < 0.01) in cells incubated with visfatin and resistin, at both tested concentrations, when compared to un-treated cells (Figure 2B).

### 2.4. Visfatin and Resistin Regulate Oxidant/Antioxidant Balance

**Figure 3:**
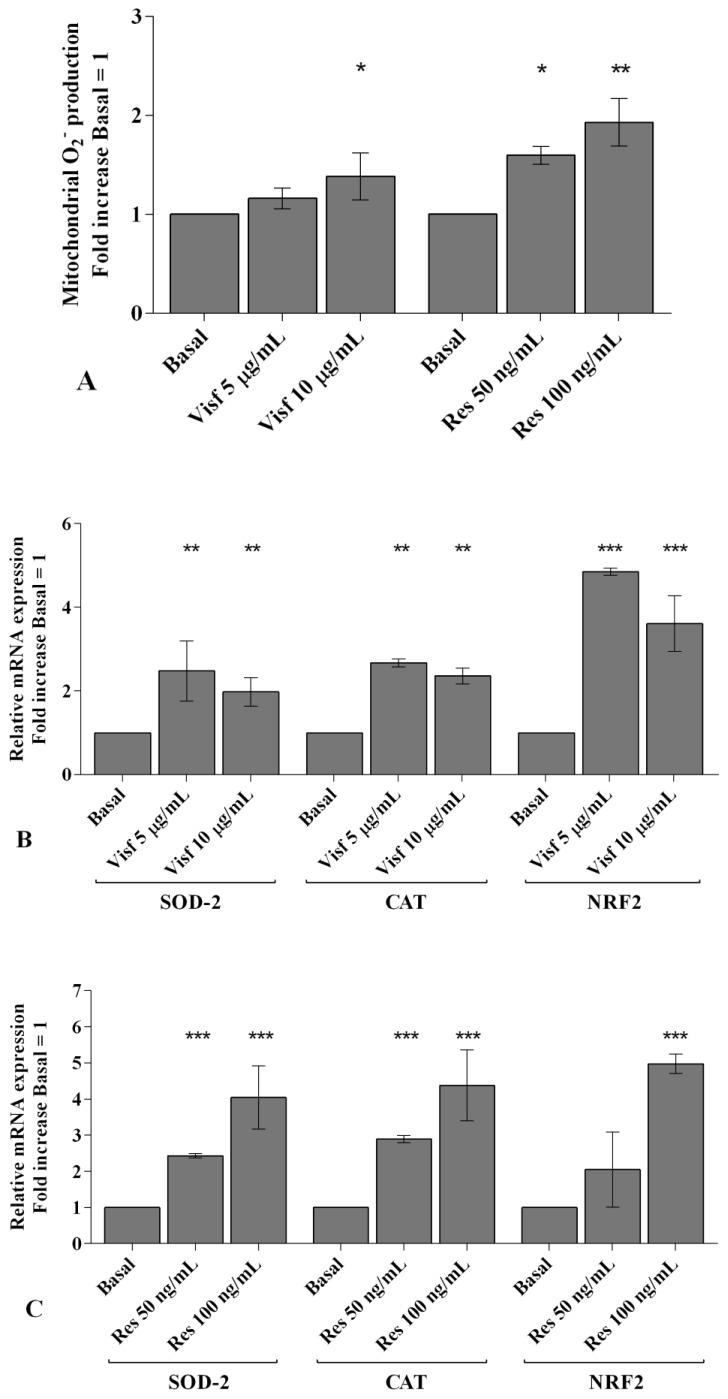
(**A**) Mitochondrial superoxide anion production was assessed by the analysis at flow cytometry using MitoSox Red staining. (**B**,**C**) Expression levels of superoxide dismutase (*SOD-2*), catalase (*CAT*), nuclear factor erythroid 2 like 2 (*NRF2*) by real-time PCR. Human osteoarthritic (OA) synovial fibroblasts were evaluated at basal condition and after incubation with visfatin (5 and 10 μg/mL) and resistin (50 and 100 ng/mL) for 24 h. The superoxide anion production and the gene expression were referenced to the ratio of the value of interest and the value of basal condition (basal, cells without treatment), reported equal to 1. Data were expressed as mean ± SD of triplicate values. * *p* < 0.05, ** *p* < 0.01, *** *p* < 0.001 versus basal condition. Visf = visfatin, Res = resistin.

To investigate the potential role of the studied adipokines in the regulation of oxidant/antioxidant balance, we assessed the production of superoxide anion and the analysis of the gene expression of the main antioxidant enzymes implicated in ROS scavenge (Figure S3 and Figure 3).

The stimulus of the cells with the higher concentration of visfatin (10 μg/mL) caused a significant increase of mitochondrial superoxide anion production (*p* < 0.05, Figure 3A); resistin 50 and 100 ng/mL significantly induced a dose-dependent activation of oxidative stress condition (*p* < 0.05, *p* < 0.01, respectively) in comparison to basal time (Figure 3A).

Both concentrations of the tested adipokines significantly up-regulated the expression levels of the antioxidant enzymes *SOD-2* (*p* < 0.01, *p* < 0.001), *CAT* (*p* < 0.01, *p* < 0.001), and *NRF2* (*p* < 0.001) (Figure 3B,C).

### 2.5. Visfatin and Resistin Modulate miRNA Gene Expression 

**Figure 4:**
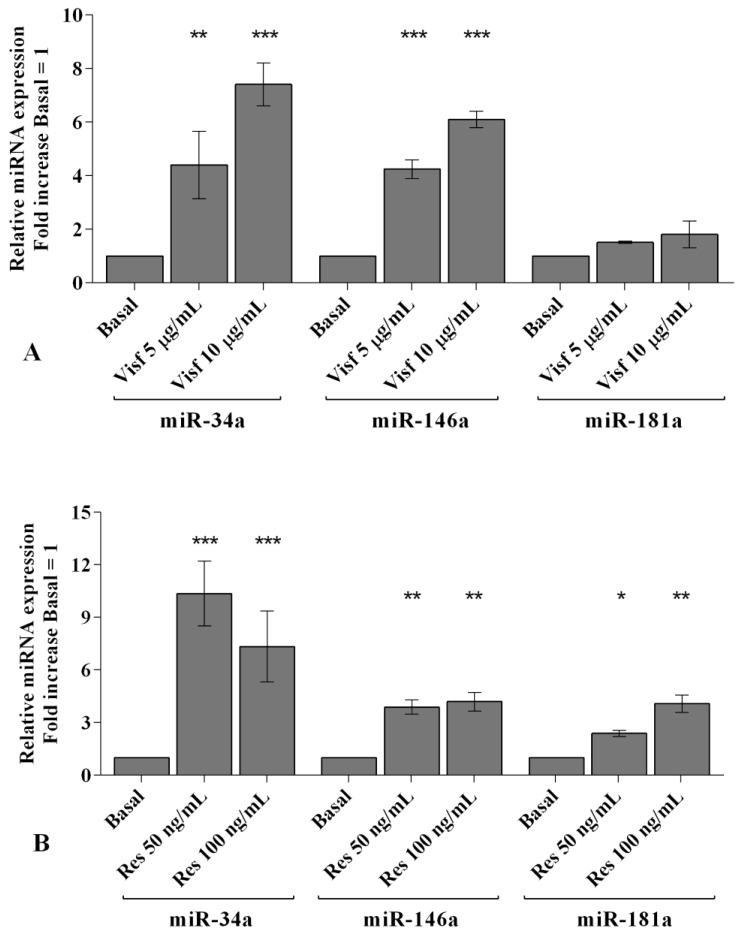
(**A**,**B**) Expression levels of *miR-34a*, *miR-146a*, and *miR-181a* by real-time PCR. Human osteoarthritic (OA) synovial fibroblasts were evaluated at basal condition and after incubation with visfatin (5 and 10 μg/mL) and resistin (50 and 100 ng/mL) for 24 h. The gene expression was referenced to the ratio of the value of interest and the value of basal condition (basal, cells without treatment), reported equal to 1. Data were expressed as mean ± SD of triplicate values. * *p* < 0.05, ** *p* < 0.01, *** *p* < 0.001 versus basal condition. Visf = visfatin, Res = resistin.

A real-time PCR analysis has been performed in order to evaluate the modulation of *miR-34a*, *miR-146a*, and *miR-181a* gene expression induced by adipokines. Visfatin at a concentration of 5 and 10 μg/mL (*p* < 0.01, *p* < 0.001) up-regulated *miR-34a* and *miR-146a* transcriptional levels in comparison to basal condition, while it did not influence *miR-181a* levels (Figure 4A). Resistin 50 and 100 ng/mL significantly increased the gene expression of *miR-34a* (*p* < 0.001), *miR-146a* (*p* < 0.01), and *miR-181a* (*p* < 0.05, *p* < 0.01) (Figure 4B).

### 2.6. MiRNA Regulate Oxidative Stress Induced by Visfatin and Resistin

**Figure 5:**
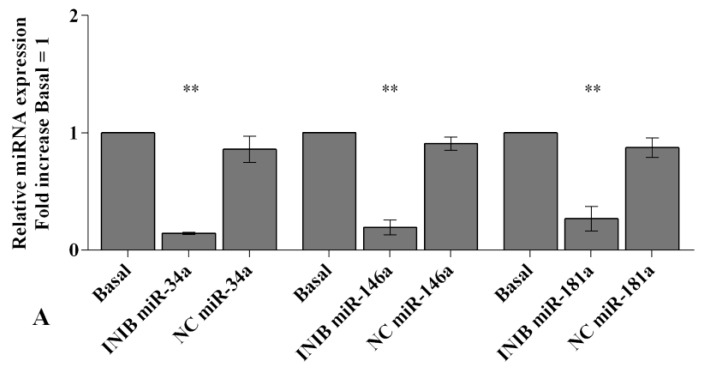

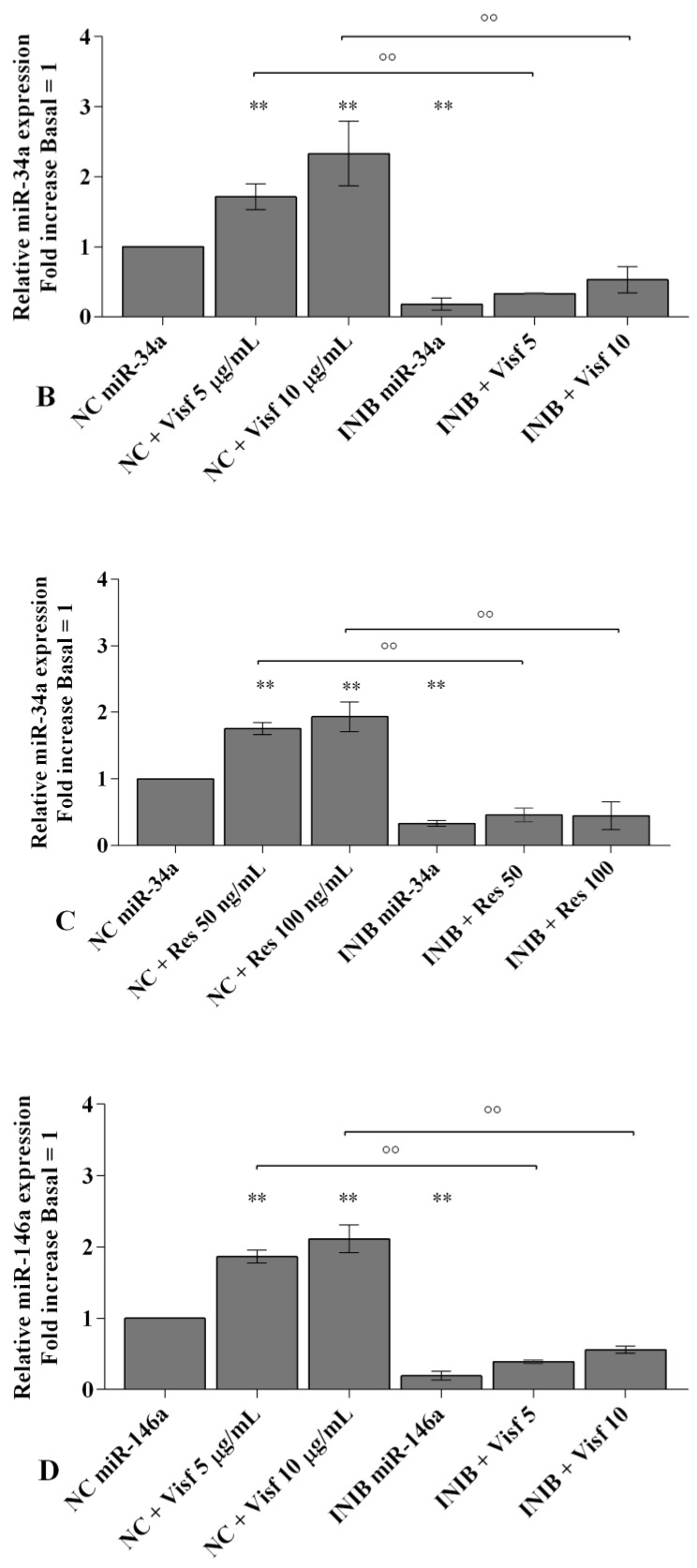

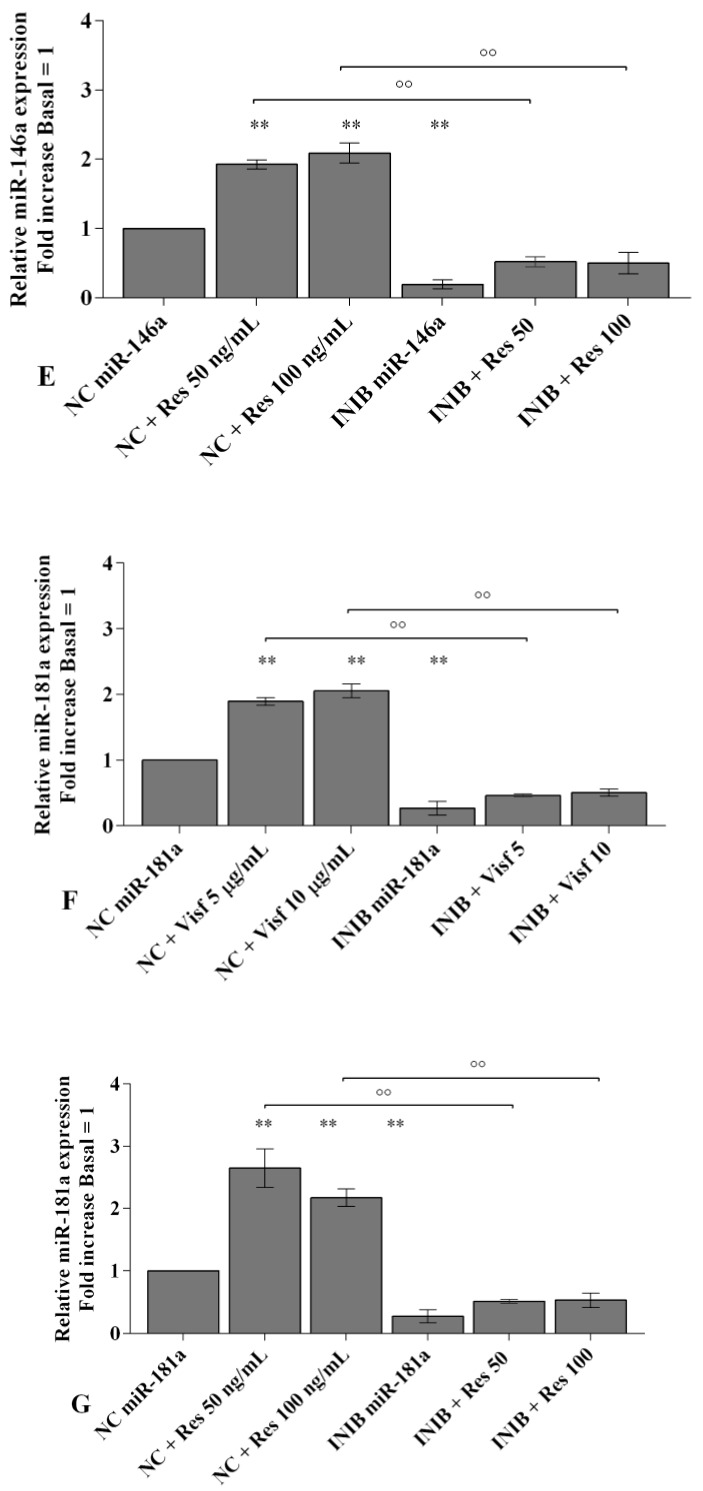
(**A**–**G**) Expression levels of *miR-34a*, *miR-146a*, and *miR-181a* by real-time PCR. Human osteoarthritic (OA) synovial fibroblasts were evaluated at basal condition, after 24 h of transfection with *miR-34a, miR-146a*, and *miR-181a* inhibitors or NC, and after incubation with visfatin (5 and 10 μg/mL) and resistin (50 and 100 ng/mL). The gene expression was referenced to the ratio of the value of interest and the value of basal condition (basal, cells without treatment) or NC, reported equal to 1. Data were expressed as mean ± SD of triplicate values. ** *p* < 0.01 versus basal condition or NC. °° *p* < 0.01 versus inhibitor. INIB= inhibitor, NC= negative control siRNA, Visf= visfatin, Res = resistin.

To confirm the involvement of miRNA in modulating oxidative stress induced by visfatin and resistin, we transfected OA synoviocytes with *miR-34a, miR-146a,* and *miR-181a* specific inhibitors (Figure 5). 

Real-time PCR showed a significant reduction of gene expression levels of the studied miRNA (*p* < 0.01) in transfected OA cells with respect to basal condition and NC (Figure 5A). 

Visfatin (5 and 10 μg/mL) and resistin (50 and 100 ng/mL) significantly up-regulated transcriptional levels of *miR-34a*, *miR-146a*, and *miR-181a* (*p* < 0.01, Figure 5B–G) in OA synoviocytes incubated with NC. After the transfection with miRNA inhibitors, the treatment with visfatin or resistin did not show any significant modification in *miR-34a*, *miR-146a*, and *miR-181a* expression in comparison to what is observed in synoviocytes transfected with the inhibitors alone (Figure 5B–G). In addition, the inhibition of *miR-34a, miR-146a*, and *miR-181a* significantly reduced the increase of miRNA transcriptional levels induced by visfatin and resistin incubation (*p* < 0.01, Figure 5B–G).

**Figure 6:**
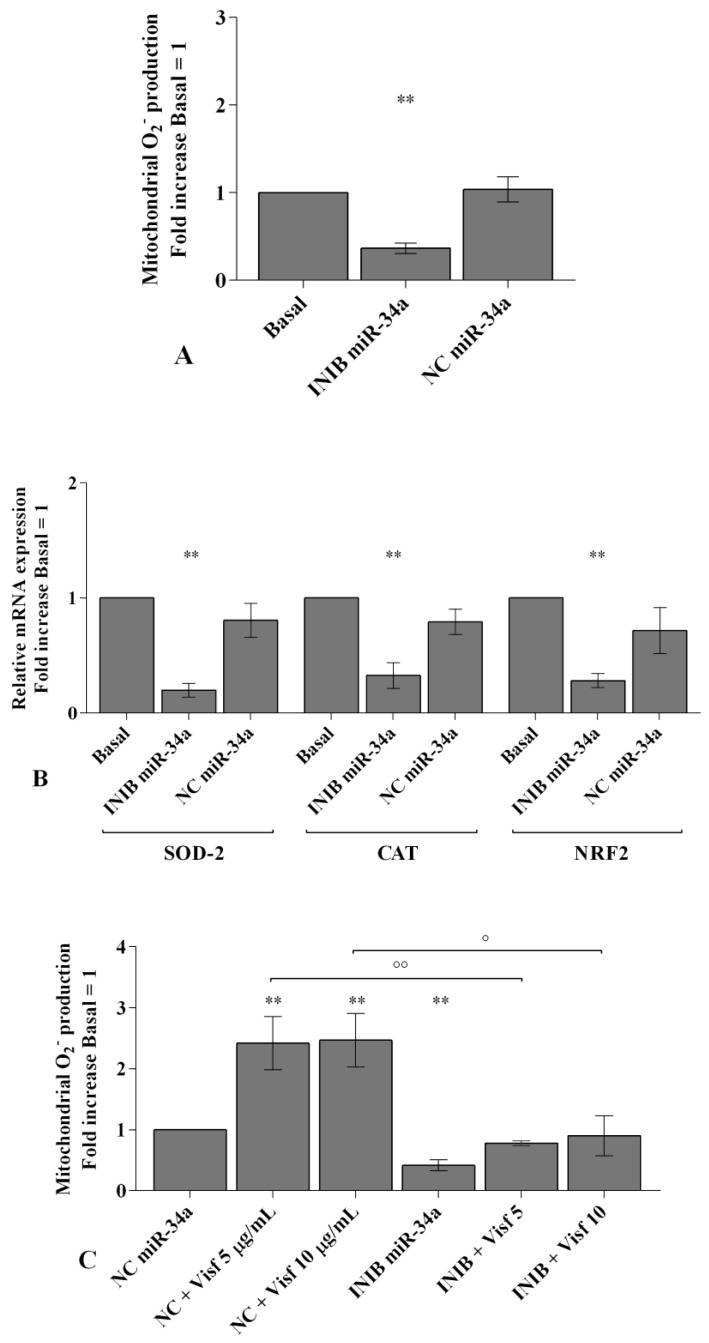

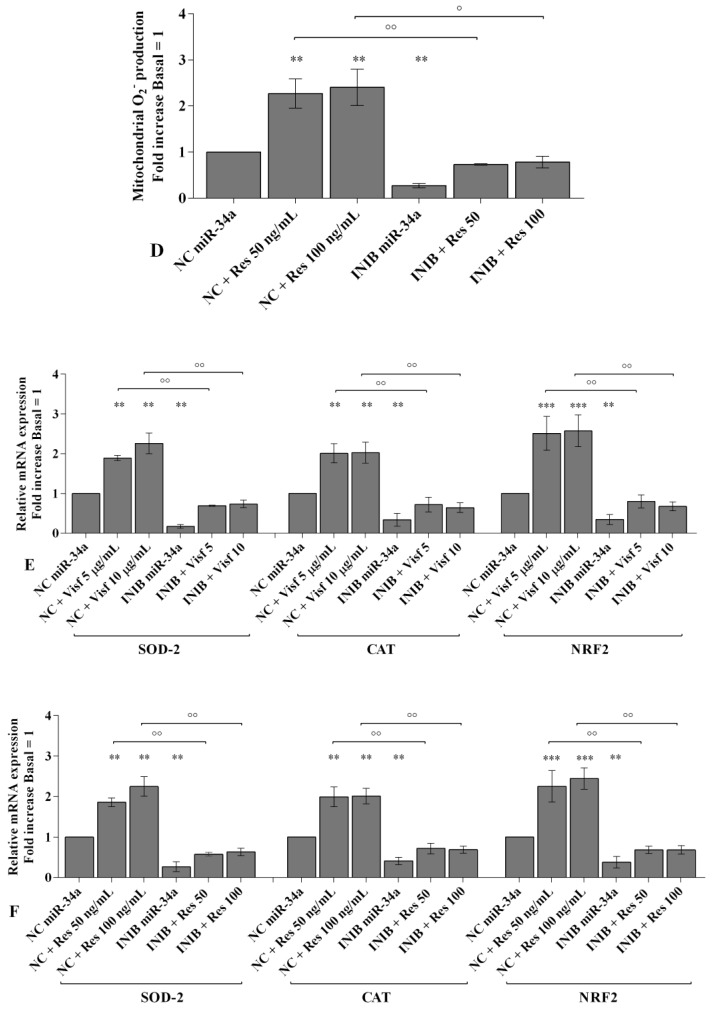
(**A**,**C**,**D**) Mitochondrial superoxide anion production was assessed by the analysis at flow cytometry using MitoSox Red staining. (**B**,**E**,**F**) Expression levels of superoxide dismutase (*SOD-2*), catalase (*CAT*), nuclear factor erythroid 2 like 2 (*NRF2*) by real-time PCR. Human osteoarthritic (OA) synovial fibroblasts were evaluated at basal condition, after 24 h of transfection with *miR-34a* inhibitor or NC, and after incubation with visfatin (5 and 10 μg/mL) and resistin (50 and 100 ng/mL). The superoxide anion production and the gene expression were referenced to the ratio of the value of interest and the value of basal condition (basal, cells without treatment) or NC, reported equal to 1. Data were expressed as mean ± SD of triplicate values. ** *p* < 0.01, *** *p* < 0.001 versus basal condition or NC. ° *p* < 0.05, °° *p* < 0.01 versus inhibitor. INIB= inhibitor, NC= negative control siRNA, Visf= visfatin, Res = resistin.

**Figure 7:**
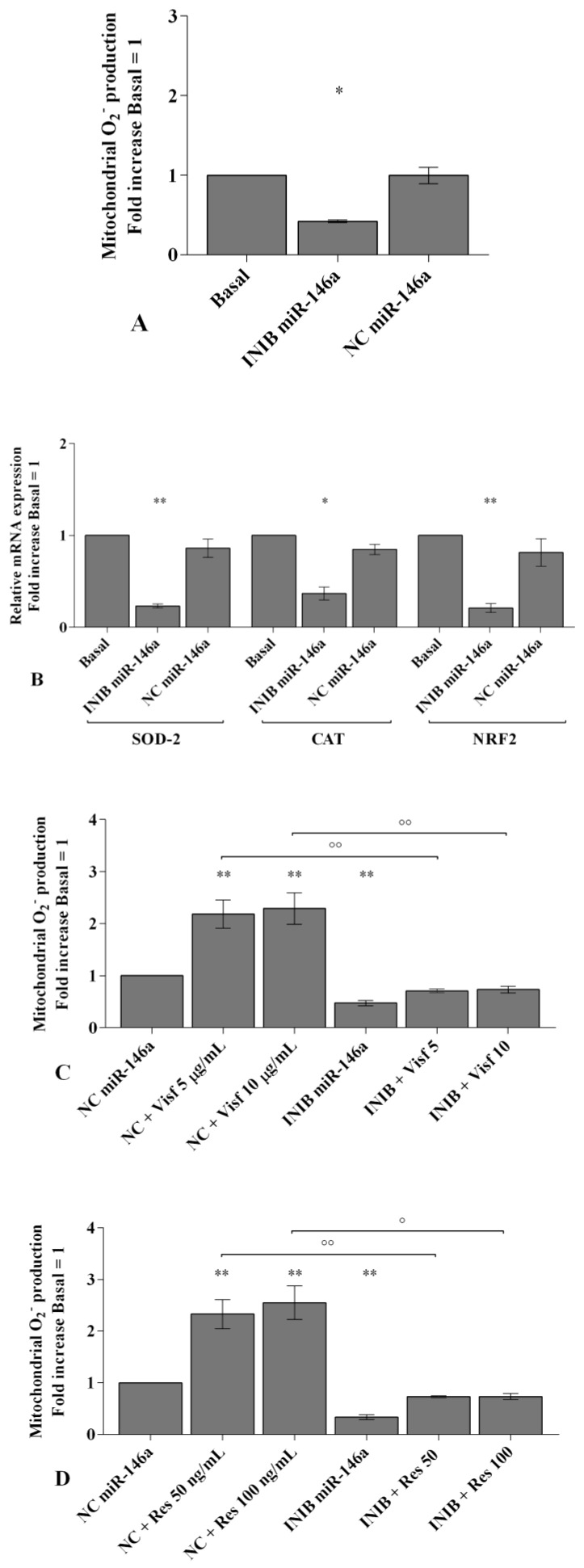

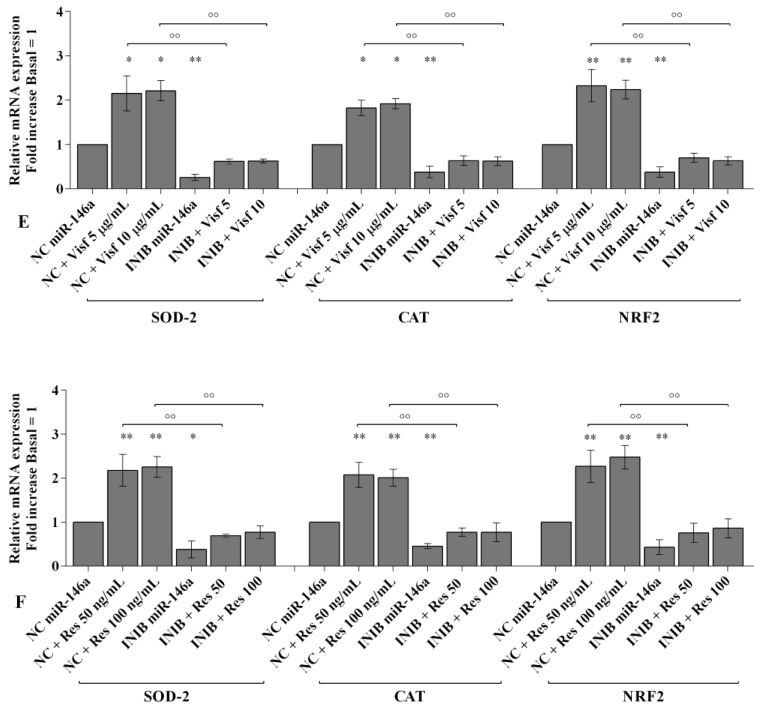
(**A**,**C**,**D**) Mitochondrial superoxide anion production was assessed by flow cytometry using MitoSox Red staining. (**B**,**E**,**F**) Expression levels of superoxide dismutase (*SOD-2*), catalase (*CAT*), nuclear factor erythroid 2 like 2 (*NRF2*) by real-time PCR. Human osteoarthritic (OA) synovial fibroblasts were evaluated at basal condition, after 24 h of transfection with *miR-146a* inhibitor or NC, and after incubation with visfatin (5 and 10 μg/mL) and resistin (50 and 100 ng/mL). The superoxide anion production and the gene expression were referenced to the ratio of the value of interest and the value of basal condition (basal, cells without treatment) or NC, reported equal to 1. Data were expressed as mean ± SD of triplicate values. * *p* < 0.05, ** *p* < 0.01 versus basal condition or NC. ° *p* < 0.05, °° *p* < 0.01 versus inhibitor. INIB= inhibitor, NC= negative control siRNA, Visf= visfatin, Res = resistin.

**Figure 8:**
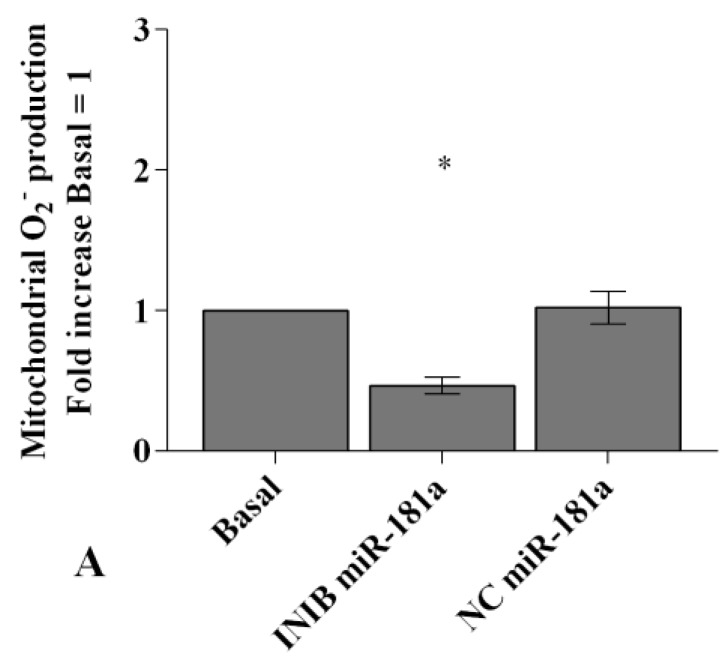

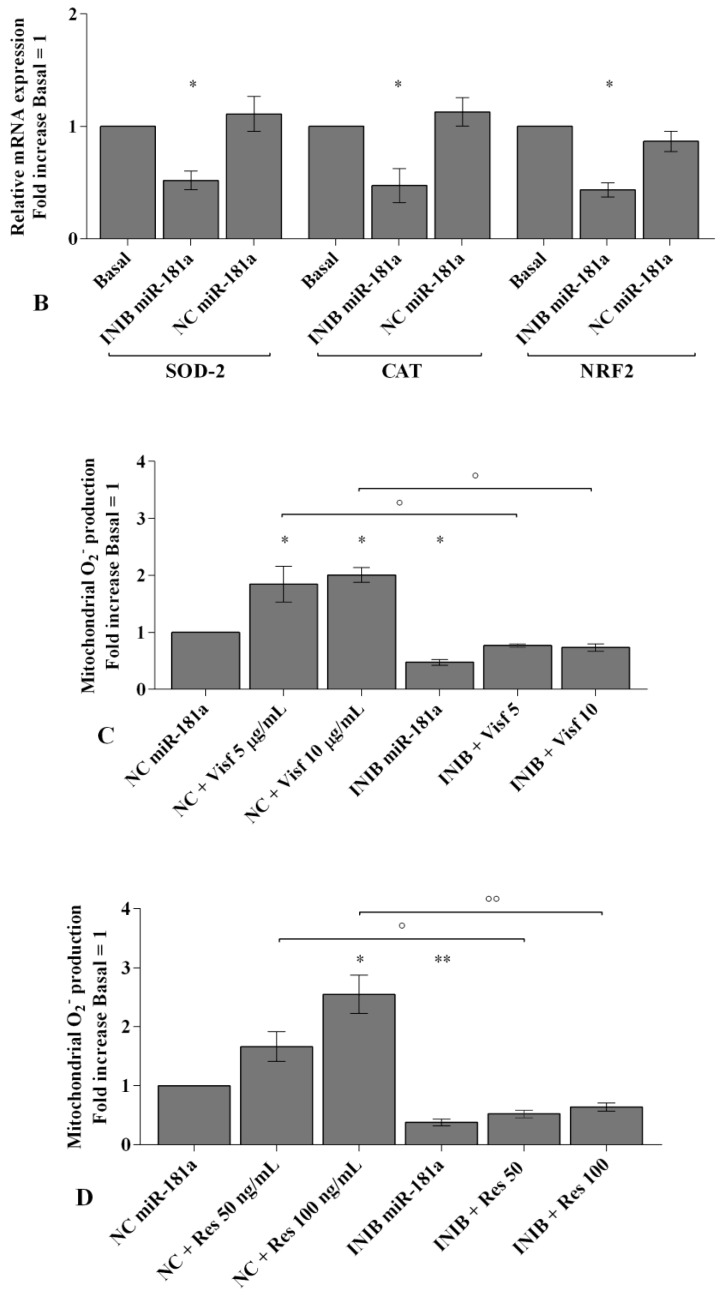

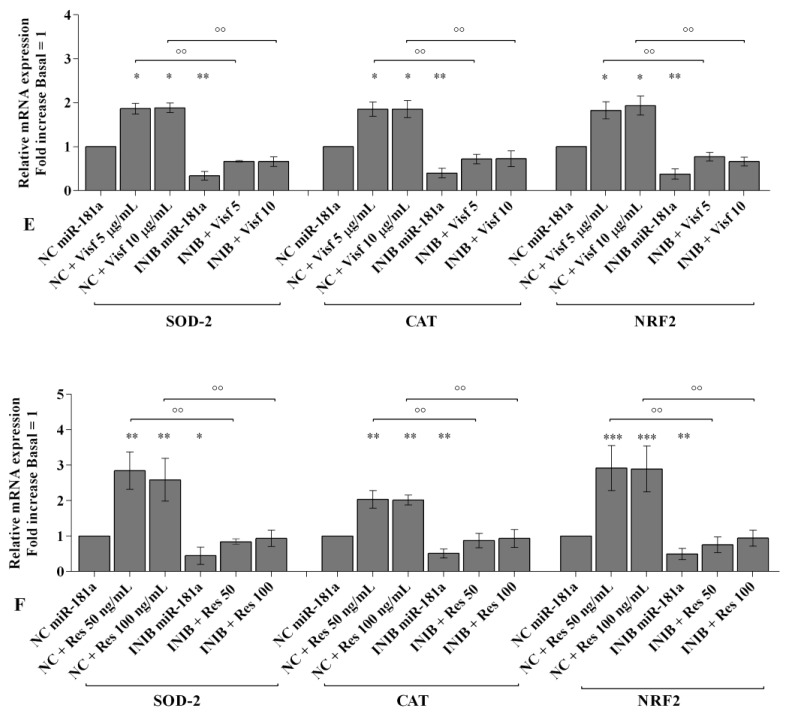
(**A**,**C**,**D**) Mitochondrial superoxide anion production was assessed by flow cytometry using MitoSox Red staining. (**B**,**E**,**F**) Expression levels of superoxide dismutase (*SOD-2*), catalase (*CAT*), nuclear factor erythroid 2 like 2 (*NRF2*) by real-time PCR. Human osteoarthritic (OA) synovial fibroblasts were evaluated at basal condition, after 24 h of transfection with *miR-181a* inhibitor or NC, and after incubation with visfatin (5 and 10 μg/mL) and resistin (50 and 100 ng/mL). The superoxide anion production and the gene expression were referenced to the ratio of the value of interest and the value of basal condition (basal, cells without treatment) or NC reported equal to 1. Data were expressed as mean ± SD of triplicate values. * *p* < 0.05, ** *p* < 0.01, *** *p* < 0.001 versus basal condition or NC. ° *p* < 0.05, °° *p* < 0.01 versus inhibitor. INIB= inhibitor, NC= negative control siRNA, Visf= visfatin, Res = resistin.

In Figure 6, Figure 7 and Figure 8 we reported the modulation of redox balance induced by visfatin and resistin after the transfection of OA synoviocytes with *miR-34a, miR-146a*, and *miR-181a* inhibitors. 

MiRNA silencing determined a significant reduction of mitochondrial superoxide anion production (*p* < 0.05, *p* < 0.01, Figure 6A, Figure 7A and Figure 8A) as well as a down-regulation of *SOD-2*, *CAT*, and *NRF2* expression levels (*p* < 0.05, *p* < 0.01, Figure 6B, Figure 7B and Figure 8B) in comparison to basal condition and NC.

The production of superoxide anion and the expression of *SOD-2*, *CAT*, and *NRF2* were increased, in a significant manner, in OA cells transfected with NC after stimulus with visfatin (*p* < 0.01, *p* < 0.001, Figure 6C,E, Figure 7C,E and Figure 8C,E) and resistin (*p* < 0.01, *p* < 0.001, Figure 6D,F, Figure 7D,F and Figure 8D,F), while their effect was significantly inhibited by *miR-34a, miR-146a*, and *miR-181a* specific inhibitors (*p* < 0.01, Figure 6C–F, Figure 7C–F and Figure 8C–F).

### 2.7. Visfatin and Resistin Activate NF-κB Signaling Pathway

**Figure 9:**
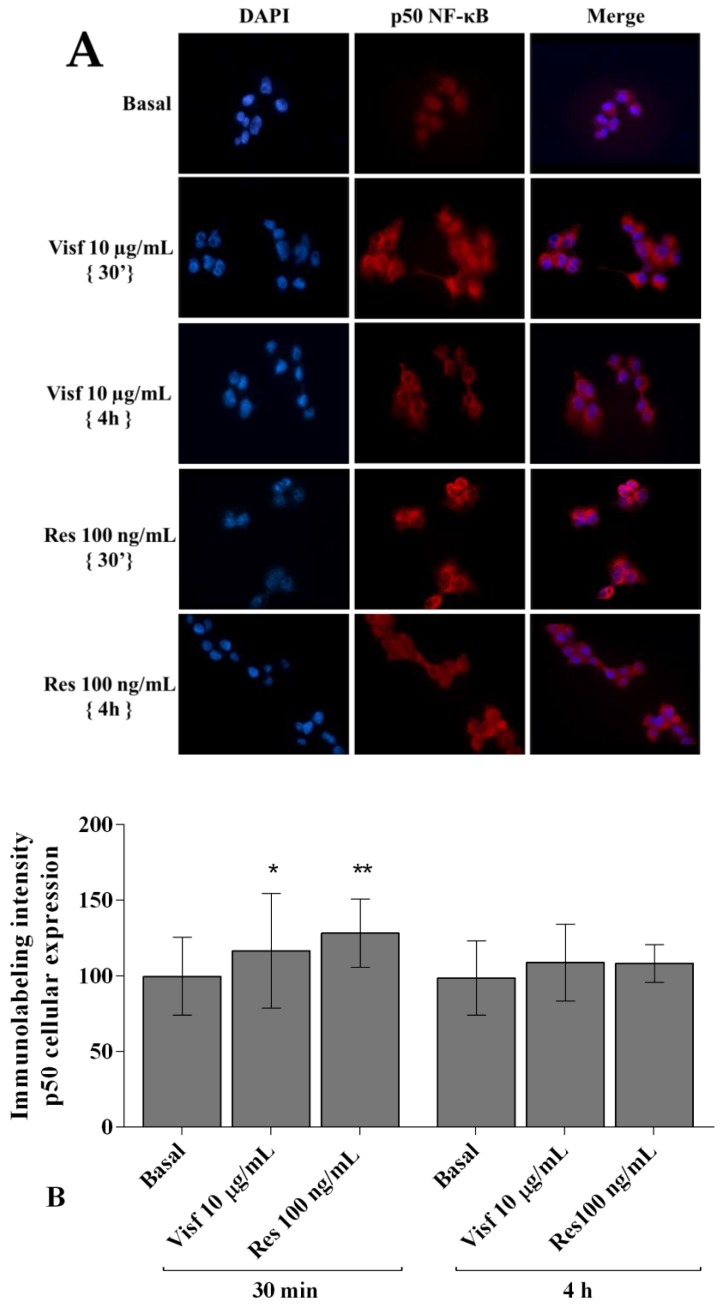
Immunofluorescence labeling of p50 NF-κB subunit localization. Human osteoarthritic (OA) synovial fibroblasts were evaluated at basal condition and after 30 min or 4 h of incubation with visfatin (10 μg/mL) and resistin (100 ng/mL). (**A**) Representative immunocytochemical images of the cells showing localization of p50 NF-κB (red); nuclei were stained with DAPI (blue). Original Magnification 400×. Scale bar: 20 μm. (**B**) The histogram of immunolabeling intensity was plotted for the nuclear and cytoplasmic expression for p50 subunit. Data were expressed as mean ± SD of triplicate values. * *p* < 0.05, ** *p* < 0.01 versus basal condition. Visf = visfatin, Res = resistin.

Figure 9A,B shows the cytoplasmic and nuclear signal intensity of p50 NF-κB subunit in synovial fibroblasts stimulated with visfatin and resistin for 30 min and 4 h. The signal of p50 NF-κB was low mainly detected in the cytoplasm of the cells, with a minimum translocation into the nucleus, at basal condition. After 30 min of incubation with visfatin and resistin we observed a significant increase of p50 subunit cytoplasmic synthesis and nuclear translocation (*p* < 0.05, *p* < 0.01, respectively), in comparison to baseline, while no significant modifications of p50 subunit signal were found after 4 h of adipokines incubation.

### 2.8. NF-κB Signaling Pathway Inhibits Visfatin and Resistin Effects 

**Figure 10:**
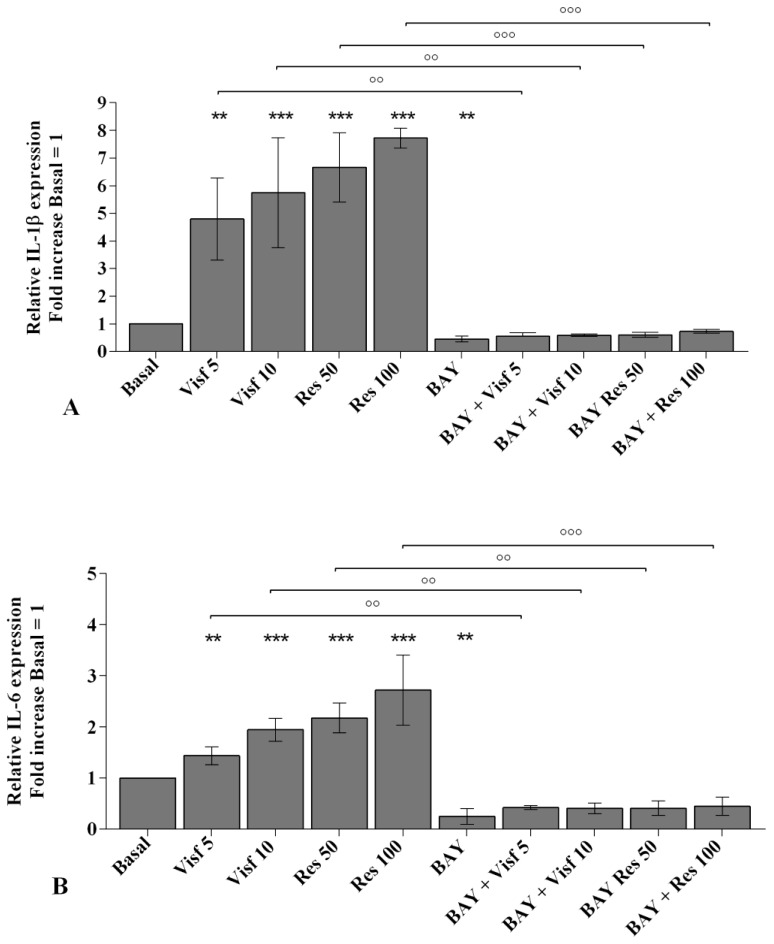

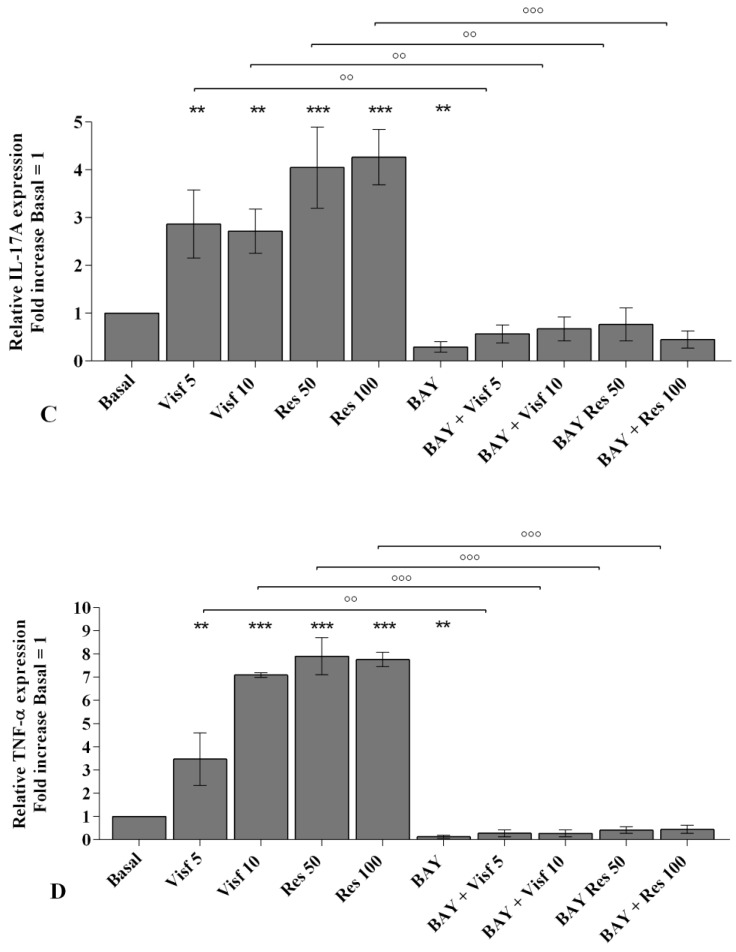
Expression levels of interleukin (*IL*)-*1β* (**A**), *IL-6* (**B**), *IL-17A* (**C**), tumor necrosis factor (*TNF*)-*α* (**D**) by real-time PCR. Human osteoarthritic (OA) synovial fibroblasts were evaluated at basal condition, after 2 h pre-incubation with a specific nuclear factor (NF)-κB inhibitor (BAY 11-7082, IKKα/β, 1 μM) and after 24 h of stimulus with visfatin (5 and 10 μg/mL) and resistin (50 and 100 ng/mL). The gene expression was referenced to the ratio of the value of interest and the value of basal condition (basal, cells without treatment) reported equal to 1. Data were expressed as mean ± SD of triplicate values, ** *p* < 0.01, *** *p* < 0.001 versus basal condition. °° *p* < 0.01, °°° *p* < 0.001 versus BAY. BAY = BAY 11-7082, Visf = visfatin, Res= resistin.

The involvement of NF-κB pathway in mediating the adipokines-induced effects on inflammatory, apoptotic and oxidative stress mediators is summarized in Figure 10.

**Figure 11:**
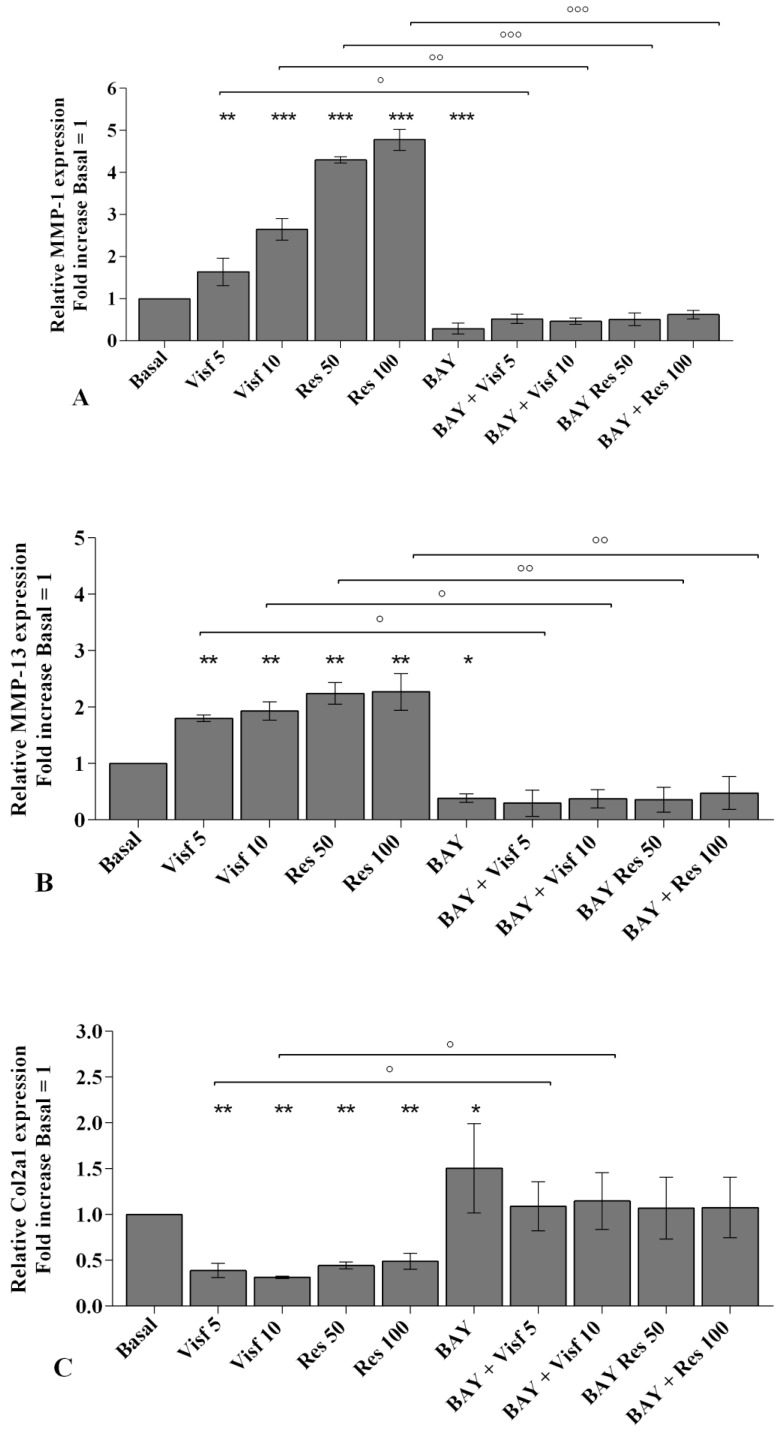
Expression levels metalloproteinases (*MMP*)-*1* (**A**), *MMP-13* (**B**), collagen type II (*Col2a1*) (**C**) by real-time PCR. Human osteoarthritic (OA) synovial fibroblasts were evaluated at basal condition, after 2 h pre-incubation with a specific nuclear factor (NF)-κB inhibitor (BAY 11-7082, IKKα/β, 1 μM) and after 24 h of stimulus with visfatin (5 and 10 μg/mL) and resistin (50 and 100 ng/mL). The gene expression was referenced to the ratio of the value of interest and the value of basal condition (basal, cells without treatment) reported equal to 1. Data were expressed as mean ± SD of triplicate values. * *p* < 0.05, ** *p* < 0.01, *** *p* < 0.001 versus basal condition. ° *p* < 0.05, °° *p* < 0.01, °°° *p* < 0.001 versus BAY. BAY = BAY 11-7082, Visf = visfatin, Res= resistin.

**Figure 12:**
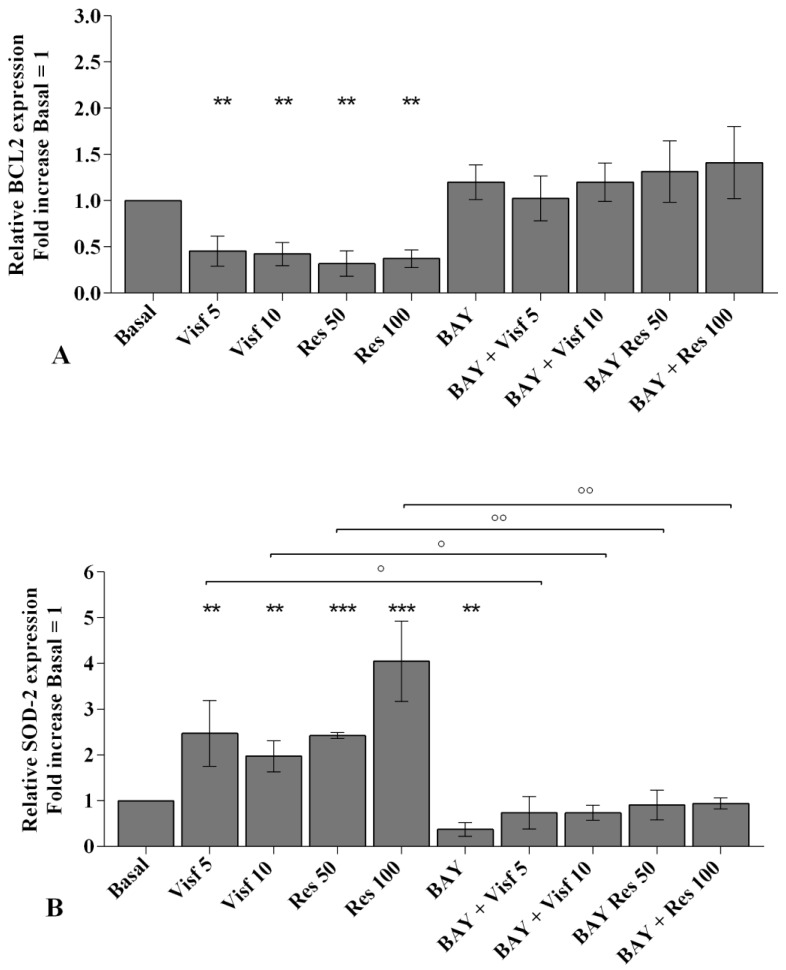

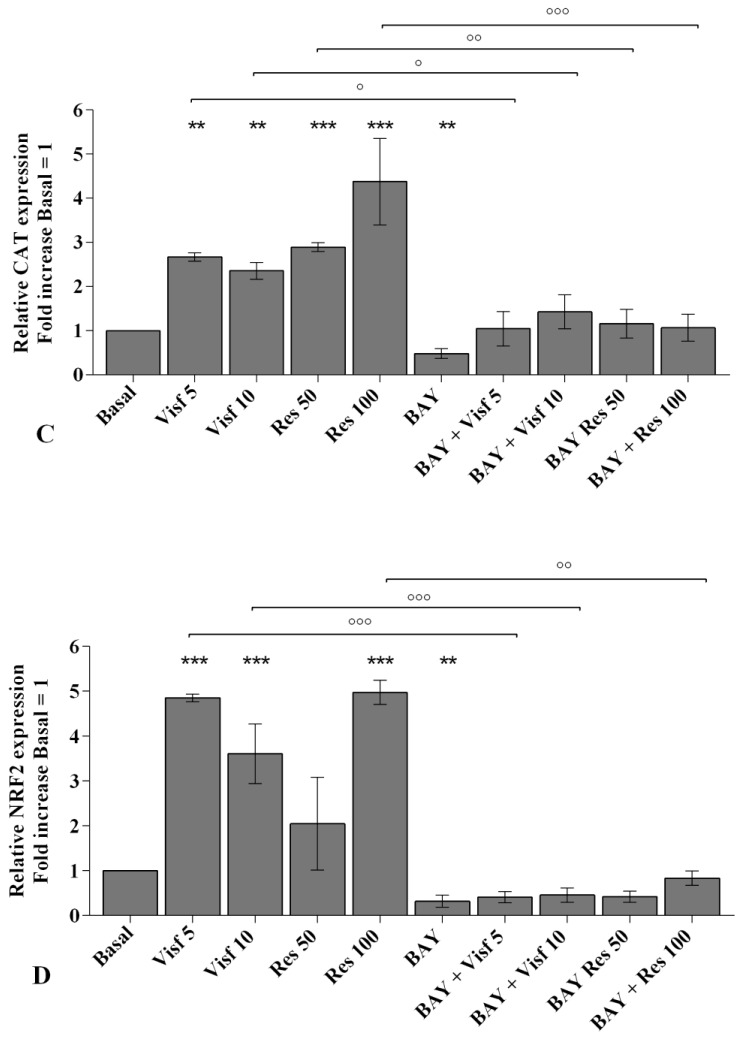
Expression levels of B-cell lymphoma (*BCL*)*2* (**A**), superoxide dismutase (*SOD-2*) (**B**), catalase (*CAT*) (**C**), nuclear factor erythroid 2 like 2 (*NRF2*) (**D**) by real-time PCR. Human osteoarthritic (OA) synovial fibroblasts were evaluated at basal condition, after 2 h pre-incubation with a specific nuclear factor (NF)-κB inhibitor (BAY 11-7082, IKKα/β, 1 μM) and after 24 h of stimulus with visfatin (5 and 10 μg/mL) and resistin (50 and 100 ng/mL). The gene expression was referenced to the ratio of the value of interest and the value of basal condition (basal, cells without treatment) reported equal to 1. Data were expressed as mean ± SD of triplicate values. ** *p* < 0.01, *** *p* < 0.001 versus basal condition. ° *p* < 0.05, °° *p* < 0.01, °°° *p* < 0.001 versus BAY. BAY = BAY 11-7082, Visf = visfatin, Res= resistin.

**Figure 13:**
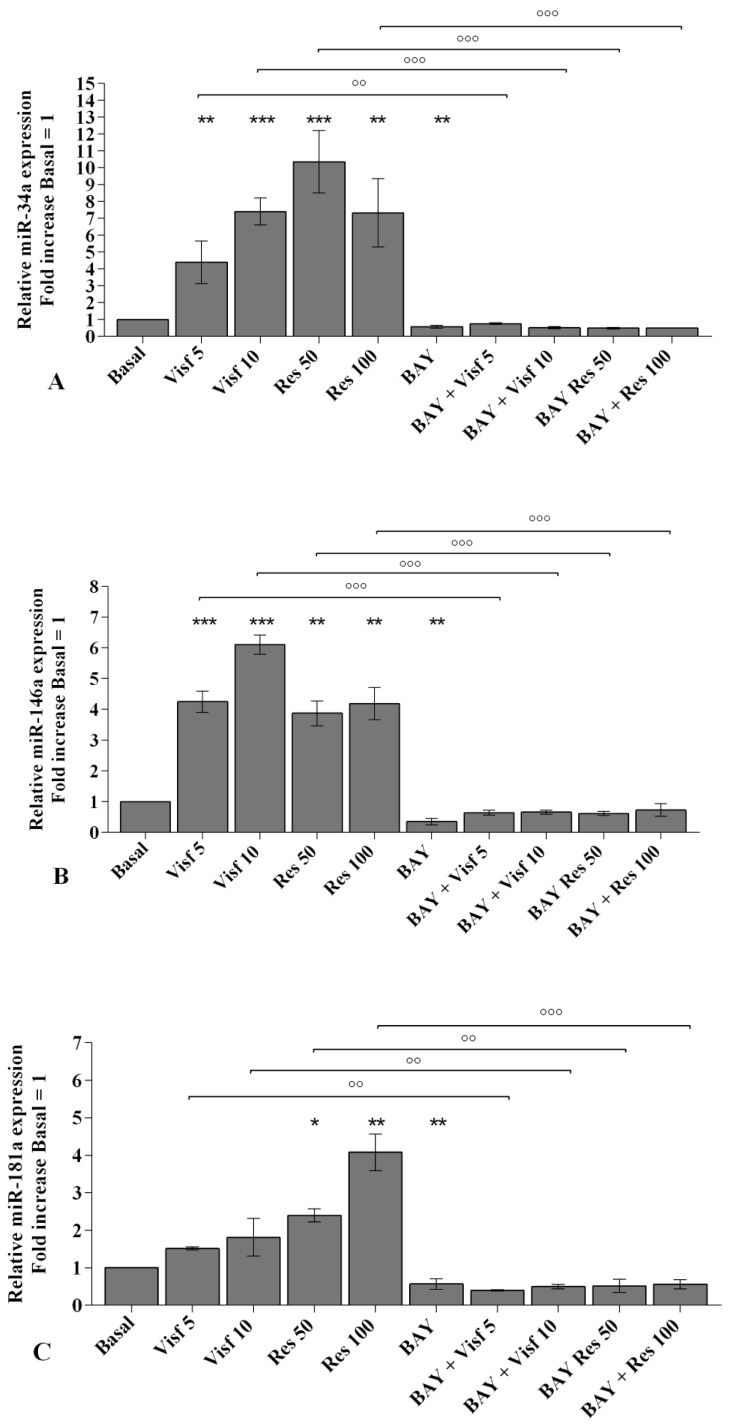
Expression levels of *miR-34a* (**A**), *miR-146a* (**B**), and *miR-181a* (**C**) by real-time PCR. Human osteoarthritic (OA) synovial fibroblasts were evaluated at basal condition, after 2 h pre-incubation with a specific nuclear factor (NF)-κB inhibitor (BAY 11-7082, IKKα/β, 1 μM) and after 24 h of stimulus with visfatin (5 and 10 μg/mL) and resistin (50 and 100 ng/mL). The gene expression was referenced to the ratio of the value of interest and the value of basal condition (basal, cells without treatment) reported equal to 1. Data were expressed as mean ± SD of triplicate values. * *p* < 0.05, ** *p* < 0.01, *** *p* < 0.001 versus basal condition. °° *p* < 0.01, °°° *p* < 0.001 versus BAY. BAY = BAY 11-7082, Visf = visfatin, Res= resistin.

A specific NF-κB inhibitor (IKKα/β, BAY 11-7082) was used to analyze the modulation of the signaling pathway in the gene expression of selected target genes (Figure 10, Figure 11 and Figure 12) and the studied miRNA (Figure 13).

The transcriptional levels of *IL-1β, IL-6, IL-17A, TNF-α* (Figure 10A–D), *MMP-1, MMP-13* (Figure 11A,B), *SOD-2*, *CAT*, *NRF2* (Figure 12B–D), *miR-34a*, *miR-146a*, and *miR-181a* (Figure 13A–C) were significantly decreased (*p* < 0.01, *p* < 0.001) in OA synovial fibroblasts incubated with BAY 11-7082, while an up-regulation of *Col2a1* mRNA levels was observed (*p* < 0.05, Figure 11C), in comparison to basal condition.

The co-treatment of the cells with BAY 11-7082 and visfatin or resistin did not exhibit any difference in miRNA and target genes expression with respect to what is observed in OA synoviocytes incubated with BAY 11-7082 alone (Figure 10, Figure 11, Figure 12 and Figure 13).

Furthermore, the pre-treatment of the cells with the NF-κB inhibitor significantly limited the effect of visfatin and resistin on the expression levels of the analyzed target genes (Figure 10, Figure 11, Figure 12 and Figure 13).

No modifications in mRNA levels of *BCL2*, after the treatment, were observed (Figure 12A). 

## 3. Discussion

OA is a musculoskeletal condition mainly characterized by articular cartilage degeneration, however, in recent years, the role of synovial inflammation in the development and in the progression of the disease has been gradually recognized [2,4].

Fibroblast-like synoviocytes actively participate in the synovitis-structural damage cycle of OA through the production of inflammatory cytokines, including IL-6, IL-1β, and TNF-α, and cartilage-degrading enzymes and proteases, such as MMPs [2,29].

Growing evidence demonstrated that adipokines, mainly produced by adipose tissue and by other adipose tissue depots as infrapatellar fat pad, are potentially involved in OA pathophysiology [30]. Indeed, the adipokines may participate in synovium-bone and synovium-cartilage interactions [7,31], however, their exact effect in OA synovial cells have not been completely elucidated [16,32,33] and the results on *in vitro* studies are sparse [19,34].

In the present study, performed in human OA synovial fibroblast cultures, we confirmed previous evidence about the role of visfatin and resistin in inflammation. Furthermore, we demonstrated their impact on apoptosis and oxidative stress processes, as well as in the modulation of some miRNA and target genes, implicated in OA pathogenesis, through the activation of NF-κB pathway. Finally, we hypothesized the direct cross-talk between miRNA and adipokines in mediating oxidative stress induction, via NF-κB signaling.

It is well established that IL-1β, IL-6, IL-17, and TNF-α are the main important cytokines involved in the pathogenesis of OA [35]; they have been found elevated in serum and synovial fluid of patients with knee OA [36,37] and play synergistic effects in OA chondrocytes and synovial fibroblasts stimulating the synthesis and secretion of other cytokines and proteases [16,29].

Our data showed a significant increase of *IL-1β*, *IL-6*, and *TNF-α* gene expression levels in human OA synovial fibroblast cultures stimulated with visfatin and resistin, according to what is observed by other authors [16,18,38]. On the other hand, we demonstrated, for the first time, the up-regulation of *IL-17* expression levels induced by the studied adipokines in our cultures.

MMPs are the main proteases implicated in cartilage turnover, playing a significant role in the degradation of cartilage ECM that occur during OA damage [39]. MMP-1 and MMP-13 are expressed in chondrocytes and in synoviocytes and contribute to promoting cartilage breakdown inducing the destruction of proteoglycans and Col2a1, the major structural protein of articular ECM [40]. The exposure of OA chondrocytes and fibroblast-like synoviocytes to pro-inflammatory cytokines, such as IL-1β, and adipokines, as visfatin and resistin, determined a markedly increase of matrix-degrading enzymes and a down-regulation of *Col2a1* gene levels [16,17,29,41,42]. In agreement with the current literature we reported the up-regulation of *MMP-1*, *MMP-13* and a reduction of *Col2a1* expression levels in visfatin and resistin-stimulated OA synovial cells.

These results highlight the role of the studied adipokines in mediating the pro-inflammatory cascade in synovial cells and their consequent implication in articular cartilage destruction that occur in course of OA. Previous evidence reporting that chondrocytes and synovial cells express membrane toll-like receptors (TLRs) which are identified as putative receptors for visfatin and resistin mechanism of action. Adipokines bind to TLRs and stimulate phosphorylation of ERK/p38/mitogen-activated protein kinase (MAPK) signaling, inducing the expression of cytokines, chemokines and degrading proteases [10,14,38,43,44].

The regulation of chondrocytes and fibroblast-like synoviocytes survival is important for the maintenance of a proper cartilage and synovium structure and function [17,45]. Indeed, apoptosis is a complex multi-step process playing a critical role in maintaining the homeostasis of various tissues and cells, and an increasing number of genes have been identified as controller and inductors of this mechanism. Among them, BCL-2 family, anti-apoptotic proteins, are responsible for many biochemical processes driving apoptosis [45].

Dysregulation of apoptosis, thus, is related to a variety of diseases including autoimmune and degenerative disorders as rheumatoid arthritis (RA) and OA [45,46]. The over-expression of BCL-2 family proteins protects OA chondrocytes and human synovial fibroblasts from the programmed cell death [47,48]. 

The results of our research revealed an increased percentage of apoptosis and a down-regulation of *BCL-2* gene expression in human OA synovial fibroblasts stimulated with visfatin and resistin. Similar data were previously obtained by other authors in endothelial cell lines and in human OA chondrocyte cultures [17,49]. However, we first observed the effect of resistin in the regulation of BCL-2 protein in this cell type.

Oxidative stress and inflammation have been increasingly recognized as being closely integrated with OA pathology. Under physiological conditions, the production of endogenous ROS is balanced by the antioxidant defense system, mainly controlled by NRF2 [50]. The latter is translocated to the nucleus, when released from its repressive cytosolic protein Kelch-like ECH associated protein 1 (KEAP1), and activates the expression of cytoprotective genes, including enzymes involved in the biosynthesis, activity, and detoxification of different ROS species, such as SOD-2 and CAT [50,51]. Various inflammatory mediators, such as cytokines, chemokines, prostaglandins, and growth factors participate to increase oxidative stress in the joint with accumulation of ROS, and nitric oxide (NO), and concomitant failure in the expression of antioxidant scavenging systems [50]. At the cellular level, oxidative stress causes mitochondrial and nuclear DNA damage, lipid peroxidation, alterations in cell signaling and transcription, and epigenetic changes in gene expression contributing to exacerbate synovitis, destruction of matrix components and cell apoptosis [50,52].

In this paper, the analysis of endogenous production of ROS reported an increase of mitochondrial superoxide anion content in OA synoviocytes cultures after visfatin and resistin stimulation, with a concomitant up-regulation of *SOD-2*, *CAT*, and *NRF2* gene expression. There is no evidence about the effects of the studied adipokines on oxidative stress induction in synovial fibroblasts; however, a number of studies, performed in different cell lines incubated with visfatin, resistin and leptin, are in agreement with our data [53,54,55].

The observed rapid increase of the studied detoxificant factors and NRF2 in adipokines-stimulated human synoviocytes confirm what is observed in a previous study on OA chondrocyte cultures [56]. In our opinion, this result could be explained as an acute adaptive response to protect mitochondria from the deleterious effects of the raised oxidant agents after adipokines stimulus [27,52,56].

Taken together, these findings underline the involvement of visfatin and resistin in the regulation of apoptosis and oxidative stress balance. This conclusion could be supported by the effects of adipokines in stimulating p38 phosphorylation to further activate PI3K/Art signaling and NADPH oxidase (NOX), a major source of ROS generation. Indeed, NOX activation cause the ROS-forming cascade signaling, induces NF-κB translocation into the nucleus, leading to likewise inflammation, cell proliferation, survival and apoptosis [53,55].

MiRNA has been widely investigated for their role in gene regulation; by binding to mRNA 3′-UTRs, miRNA can affect many protein-encoding genes at the post-transcriptional levels [22,24,57]. It is proved that some miRNA are differentially expressed in OA cartilage samples with respect to normal ones, demonstrating their role in the development and progression of OA [23,24,26].

*MiR-34a* is largely known to be an anti-proliferative factor regulating cell cycle arrest or senescence [58]. Some authors reported the involvement of *miR-34a* in activating apoptosis signaling and limiting cell proliferation in human OA chondrocytes and RA synovial fibroblasts [59,60], as well as its role in modulation of oxidative stress balance in HUVEC lines [61].

*MiR-181a* was found highly expressed in circulating PBMC of OA patients and in human OA chondrocytes [62,63], and its results implicated the regulation of apoptosis and oxidative stress signaling by targeting multiple anti-apoptotic BCL2 members and modulating mitochondria metabolism in different cell types [63,64,65].

Data from the current literature concerning the involvement of miR-146a in OA pathogenesis are controversial [27,66,67]. Yamasaki et al. [66] demonstrated that this miRNA is up-regulated in OA cartilage with a low grade on the Mankin scale, or after the stimulus of OA chondrocytes with IL-1β [67]. On the contrary, its reduced expression in hydrogen peroxide-stimulated OA cells was observed [27]. Additionally, this miRNA resulted implicated in oxidative stress regulation by its direct effect on NRF2 transcriptional factor [68].

In this study we showed a significant increase of *miR-34a*, *miR-146a*, and *miR-181a* gene expression after the incubation of OA synoviocytes with visfatin and resistin, consistently with the results of other in vitro studies [17,18,69,70,71]. On the basis of the results obtained by Wu et al. [18] we can hypothesize the modulation of miRNA gene expression through the phosphorylation of ERK/p38/MAPK signaling induced by visfatin and resistin. 

Accumulating evidence has shown a cross-talk between miRNA and components of redox signaling [27,28,57,72]. The transcription, biogenesis, translocation, and function of miRNA are highly correlated with ROS, and, meanwhile, miRNA can regulate the expression of redox factors and other ROS modulators, such as the key components of cellular antioxidant machinery [27,28,57].

Recently, some miRNA were identified as oxidative stress-responsive factors after the treatment of OA chondrocytes with H_2_O_2_ [27,73], on the other hand, cellular mechanisms regulating oxidative stress were fine-tuned by particular miRNA [28,56]. 

A number of studies demonstrated the regulation of *miR-34a*, *miR-146a*, and *miR-181a* expression by oxidative stress in PC12, cardiac and carcinoma cell lines and in OA chondrocytes [27,74,75,76]; furthermore, the inhibition of these miRNA decreased the expression of the main antioxidant enzymes and reduced the mitochondrial intracellular ROS levels [56,61,64,74,76]. According to this evidence, in the present study, the transient transfection of OA synovial fibroblasts with *miR-34a, miR-146a*, and *miR-181a* specific inhibitors significantly reduced the production of mitochondrial superoxide anion as well as the expression of *SOD-2*, *CAT*, and *NRF2*, limiting the negative effects of visfatin and resistin. In a similar manner, other authors revealed the involvement of miRNA in mediating visfatin and resistin effects in HepG2 cells and in human synovial fibroblasts [18,71]. The ability of these miRNA in regulating oxidative stress has been reported in different in vitro studies and seems to be related to the regulation of NRF2 activity [57]. Huang et al. [77] showed the implication of miR-34a in modulating *NRF2* expression and NRF2-dependent antioxidant pathway through the direct targeting of *miR-34a* with the 3′UTR of *NRF2* mRNA. Furthermore, *miR-146a* resultingly involved in the regulation of *NRF2* activation by targeting the 3′-UTR of IL-1R-associated kinase (*IRAK)1* and TNFR-associated factor (*TRAF)6* mRNA, the downstream adaptors of TLRs [68]. These data suggest the presence of a regulatory network between miRNA and NRF2 in regulating oxidative stress. 

However, in the present study we observed a reduction in the gene expression of antioxidant enzymes when the miRNA were inhibited. This finding could be due to the fact that *miR-34a* and *miR-181a* also directly bind the 3′UTR of silent mating type information regulation 2 homolog (*SIRT)1* mRNA, inducing a decrease in the protein and/or mRNA expression of this gene. 

SIRT1 and SIRT6 are putative anti-ageing molecules that regulate the expression of several antioxidant genes and are classified as regulator of oxidative stress balance. Elevated oxidative stress decreased both the protein and mRNA levels of *SIRT1*, whilst up-regulating the expression of miR-34a and miR-181a. 

In view of these reports, we can postulate that the obtained results concerning the gene expression of antioxidant enzymes could be related to the up-regulation of *SIRT1* after of *miR-34* and *miR-181a* inhibition [77]. 

We finally supposed that the complex crosstalk found between adipokines and miRNA, in OA synovial fibroblasts, could be regulated by NF-κB signaling pathway.

NF-κB proteins constitute a family of ubiquitously expressed transcription factors playing essential roles in phlogistic events, immune and stress responses, and in cartilage degradation [78,79]. Accumulation data indicate NF-κB signaling as the most prominent mechanism in the pathogenesis of OA [78,79]. Furthermore, the importance of NF-κB signaling pathway for visfatin and resistin-induced inflammation, as well as for miRNA-related post-transcriptional regulation has been reported [16,17,18,19,56,70].

Our results showed an increase of NF-κB activation and of p50 subunit nuclear translocation in OA synoviocytes stimulated with visfatin and resistin, in agreement with other researches performed in various cell cultures [16,17,33,49,55,80,81]. Besides, these studies also affirmed that NF-κB is involved in regulation of visfatin and resistin-mediated effects in human OA chondrocytes and endothelial progenitor cells incubated with a specific NF-κB inhibitor [16,33,49,55]. Our data support these findings demonstrating that the inhibition of NF-κB signaling limits inflammation and oxidative stress induced by visfatin and resistin, in human OA synovial fibroblasts. The current literature establishes the activation of NF-κB signaling after phosphorylation of ERK/p38/ MAPK pathway induced by visfatin and resistin, triggering the downstream up-regulation of pro-inflammatory and pro-catabolic-related genes, which contribute to inflammatory and degrading processes of OA. Hence, the inhibition of NF-κB transcriptional factor could represent one of the molecular mechanisms to limit adipokines effects on joint injury.

In addition, we also observed that the modulation of *miR-34a, miR-146a*, and *miR-181a* expression induced by the studied adipokines was strongly limited by NF-κB inhibition. Similar results were found by other authors, showing an increased gene expression of *miR-34a* and *miR-146a* after IL-1β stimulus through activation of NF-kB; in turn, *miR-34a* and *miR-146a* were found to be able to inhibit the activation of NF-kB via suppressing their target genes expression such as *NRF2*, *IRAK1* and *TRAF6* [68,77,82]. 

These data suggest that the cross-talk between visfatin, resistin and miRNA could be mediated by NF-κB signaling pathway, highlighting the mutual interaction between miRNA and NF-kB.

However, the present study presents some limitations that need to take into consideration. 

First of all, additional experiments on healthy primary cells are recommended; further transfection experiments with specific miRNA mimic could be useful to confirm the regulation induced by the studied miRNA. In addition, the protein levels of the antioxidant enzymes and of the transcriptional factor NRF2 should be detected as well to elucidate if transcription modifications reflect a translational regulation.

Finally, a simultaneous miRNA and NF-κB inhibition could help to deeper investigate their direct interaction in mediating adipokines effects.

## 4. Materials and Methods 

### 4.1. Sample Collection and Cell Culture

Synovial tissue samples were obtained from three non-obese (BMI from 20 to 25 Kg/m^2^) and non-diabetic patients (two men and three women, age from 67 to 75) with primary knee OA defined by the clinical and radiological ACR criteria [83], during their total knee arthroplasty. The tissues were supplied by the Orthopaedic Surgery, University of Siena, Italy. The human articular samples protocols used in this work were evaluated and approved by the Ethic Committee of Azienda Ospedaliera Universitaria Senese/Siena University Hospital (Prot n 13931_2018, 15 October 2018), and all patients signed a free and informed consent form.

Synovial tissue was separated from adjacent cartilaginous and adipose structures, and isolated immediately after surgery. Briefly, samples were aseptically dissected from each donor, cut into small thick pieces and processed by an enzymatic digestion by using trypsin-EDTA Solution 10× (Sigma–Aldrich, Milan, Italy) for 15 min at 37 °C and then, washed and incubated with type IV collagenase (Sigma–Aldrich, Milan, Italy) in Dulbecco’s Modified Eagle Medium (DMEM) (Euroclone, Milan, Italy) medium with shaking for 12–16 h at 37 °C.

The obtained cell suspension was filtered using 70-μm nylon meshes, washed, and centrifuged for 5 min at 700× *g*. The viability was assessed by Trypan Blue (Sigma–Aldrich, Milan, Italy) test and a percentage of 90% to 95% of cell survival was assessed. Cells were collected, seeded into 10-cm diameter tissue culture plates, and expanded for a minimum of two weeks in a monolayer in incubator with 5% CO_2_ and 90% humidified atmosphere at 37 °C, until a confluence of 80% to 85% was reached.

Human OA synovial fibroblasts were grown in DMEM containing 10% fetal bovine serum (FBS) (Euroclone, Milan, Italy), with 200 U/mL penicillin and 200 µg/mL streptomycin (P/S) (Sigma–Aldrich, Milan, Italy). The culture medium was changed two times for week. The morphology was examined daily with an inverted microscope (Olympus IMT-2, Tokyo, Japan), and the cells from passages 3 to 6 were employed for the experimental procedures. A cell culture derived from a unique donor was used for each single experiment, for a total of three independent experiments.

### 4.2. Stimulus of Synovial Cell Cultures

Human OA synovial fibroblasts were transferred and plated in 6-well dishes at a starting density of 1 × 10^5^ cells/well until they became confluent. Human recombinant visfatin (Sigma–Aldrich, Milan, Italy) and human recombinant resistin (BioVendor, Rome, Italy) were dissolved in phosphate buffered saline (PBS) (Euroclone, Milan, Italy), according to the manufacturer’s instructions, and then directly diluted in the culture medium for the treatment in order to obtain the final concentration required.

The cells were immersed in DMEM medium enriched with 0.5% FBS and 2% P/S and stimulated for 24 h with visfatin at concentration of 5 and 10 μg/mL or resistin 50 and 100 ng/mL. The concentrations of the adipokines used in our in vitro study were selected according to those used by other authors and in our previous report [16,17,84]; the final concentrations were chosen based on the best results obtained in terms of viability (Figure S1).

After the treatment, the cells were recovered and immediately processed to carry out flow cytometry analysis and quantitative real-time PCR.

In addition, the synovial cells were pre-incubated for 2 h with 1 μM BAY 11-7082 (NF-κB inhibitor, IKKα/β, Sigma–Aldrich, Milan, Italy) and then stimulated 24 h with the selected concentrations of visfatin (5 and 10 μg/mL) and resistin (50 and 100 ng/mL). Then, the gene expression of the target genes (*IL-1β, IL-6, IL-17A, TNF-α, MMP-1, MMP-13, Col2a1, BCL2, SOD-2, CAT* and *NRF2*) and miRNA (*miR-34a, miR-146a*, and *miR-181a*) was evaluated.

### 4.3. MTT Assay

The viability of the cells was evaluated, by MTT test, after the treatment of the cells with visfatin and resistin at the tested concentrations. 

Chondrocytes were incubated for 3 h at 37 °C in a culture medium containing 10% of 5 mg/mL of MTT (Sigma–Aldrich, Milan, Italy). At the end of this period, the medium was removed and 0.2 mL of dimethyl sulfoxide (DMSO) (Rottapharm Biotech, Monza, Italy) was added to the wells to solubilize the formazan crystals. The absorbance was measured at 570 nm in a microplate reader (BioTek Instruments, Inc., Winooski, VT, USA). A control well without cells was employed for blank measurement.

The percentage of survival cells was evaluated as (absorbance of considered sample) / (absorbance of control) × 100.

The experiments were performed on cell cultures at 80% to 85% of confluence in order to prevent contact inhibition which can alter the results. Data were reported as OD units per 10^4^ adherent cells.

### 4.4. Transfection of Synovial Cells

The cells were grown in 6-well dishes at a starting density of 1 × 10^5^ cells/well until a confluence of 85% in DMEM supplemented with 10% FBS; then, the media were replaced with DMEM 0.5% FBS for 6 h before transfection. Afterwards, synoviocytes were transfected with specific inhibitors of *miR-34a, miR-146a*, and *miR-181a* (Qiagen, Hilden, Germany), at the concentration of 50 nM, or with their relative negative controls siRNA (NC) (Qiagen, Hilden, Germany), at the concentration of 5 nM, in serum-free medium for a period of 24 h. Supernatants were removed and synoviocytes immediately harvested or incubated with visfatin (5 and 10 μg/mL) or resistin (50 and 100 ng/mL) for additional 24 h.

### 4.5. Quantitative Real-Time PCR of mRNA and miRNA

Synovial fibroblasts were grown in 6-well dishes at a starting density of 1 × 10^5^ cells/well in DMEM supplemented with 10% FBS. Then, the supernatant was removed, and the cells were cultured in DMEM with 0.5% FBS used for the treatment procedure.

Total RNA, including miRNA, was extracted using TriPure Isolation Reagent (Euroclone, Milan, Italy) according to the manufacturer’s instructions, and was stored at −80 °C. The concentration, purity, and integrity of RNA were evaluated by measuring the OD at 260 nm and the 260/280 and 260/230 ratios by Nanodrop-1000 (Celbio, Milan, Italy). The quality of RNA was verified by electrophoresis on agarose gel (FlashGel System, Lonza, Rockland, ME, USA). Reverse transcription for miRNA was carried out by the cDNA miScript PCR Reverse Transcription kit (Qiagen, Hilden, Germany), while for target genes the QuantiTect Reverse Transcription kit (Qiagen, Hilden, Germany) was used, according to the manufacturer’s instructions.

MiRNA and target genes were examined by real-time PCR using, miScript SYBR Green (Qiagen, Hilden, Germany) and QuantiFast SYBR Green PCR (Qiagen, Hilden, Germany) kits, respectively. A list of the used primers is reported in Table 1. 

All qPCR reactions were achieved in glass capillaries by a LightCycler 1.0 (Roche Molecular Biochemicals, Mannheim, Germany) with LightCycler Software Version 3.5. The reaction procedure for miRNA consisted of 95 °C for 15 min for HotStart polymerase activation, followed by 40 cycles of 15 s at 95 °C for denaturation, 30 s at 55 °C for annealing, and 30 s at 70 °C for elongation, according to the protocol. Target genes amplification was performed at 5 in at 95 °C, 40 cycles of 15 s at 95 °C, and 30 s at 60 °C. In the final step of both protocols, the temperature was raised from 60 °C to 95 °C at 0.1 °C/step to plot the melting curve.

The analysis of the dissociation curves was performed by visualizing the amplicons lengths in agarose gel to confirm the correct amplification of the resulting PCR products.

For the data analysis, the C_t_ values of each sample and the efficiency of the primer set were calculated through LinReg Software [85] and then converted into relative quantities and normalized using the Pfaffl model [86].

The normalization was performed considering Small Nucleolar RNA, C/D Box 25 (SNORD-25) for miRNA and Actin Beta (ACTB) for target genes, as the housekeeping genes. The choice of the genes was carried out by using geNorm software version 3.5 [87].

### 4.6. Apoptosis Detection

Apoptotic cells were evaluated by using Annexin V-FITC and propidium iodide (PI) (ThermoFisher Scientific, Milan, Italy). Human OA synovial fibroblasts were seeded in 12-well plates (8 × 10^4^ cells/well) for 24 h in DMEM with 10% FBS. Then, the medium was discarded, and the cells were cultured in DMEM with 0.5% FBS used for the treatment procedure. Afterwards, the synovial cells were washed and harvested by using trypsin, collected into cytometry tubes, and centrifuged at 1500 rpm for 10 min. The supernatant was replaced, and the pellet was resuspended in 100 μL of 1× Annexin-binding buffer, 5 μL of Alexa Fluor 488 annexin-V conjugated to fluorescein (green fluorescence) and 1 μL of 100 μg/mL PI working solution. Markers were added to 100 μL of cell suspension. Cells were incubated at room temperature for 15 min in the dark. Then, 600 μL of 1× Annexin-binding buffer were added before the analysis at flow cytometer. A total of 10,000 events (1 × 10^4^ cells per assay) were measured by the instrument. The obtained results were analyzed with Cell Quest software (Version 4.0, Becton Dickinson, San Jose, CA, USA). The evaluation of apoptosis was carried out considering staining cells simultaneously with Alexa Fluor 488 annexin-V and PI; a discrimination of intact cells (annexin-V and PI-negative), early apoptosis (annexin-V-positive and PI-negative), and late apoptosis (annexin-V and PI-positive) is allowed [88].

The results were expressed as percentage of positive cells to each dye (total apoptosis), and the data were represented as the mean of three independent experiments (mean ± SD).

### 4.7. Mitochondrial Superoxide Anion (•O_2_-) Production

Human OA synovial fibroblasts were seeded in a density of 8 × 10^4^ cells per well in 12 multi-plates for 24 h in DMEM with 10% FCS. Then, the medium was eliminated, and the cells were cultured in DMEM with 0.5% FBS used for the treatment procedure. Then, the cells were incubated in Hanks’ Balanced Salt Solution (HBSS) and MitoSOX Red for 15 min at 37°C in dark, to assess mitochondrial superoxide anion (•O_2_-) production. MitoSOX was dissolved in DMSO, at a final concentration of 5 µM. Cells were then harvested by trypsin and collected into cytometry tubes and centrifuged at 1500 rpm for 10 min. Besides, cells were suspended in saline solution before being analyzed by flow cytometry. A density of 1 × 10^4^ cells per assay (a total of 10,000 events) were measured by flow cytometry and data were analyzed with CellQuest software (Version 4.0, Becton Dickinson, San Jose, CA, USA). Results were collected as median of fluorescence (AU) and represented the mean of three independent experiments (mean ± SD).

### 4.8. Immunofluorescence Analysis

Human OA synovial fibroblasts were plated in coverslips in Petri dishes (35 × 10 mm) at a starting low density of 4 × 10^4^ cells/chamber, to prevent possible cell overlapping, and re-suspended in 2 mL of culture medium until 80% of confluence. The cells were processed after 2 h of stimulus with adipokines to evaluate the potential activation of the NF-κB pathway. The synovial cells were washed in PBS and then fixed in 4% paraformaldehyde (ThermoFisher Scientific, Milan, Italy) (pH 7.4) for 10 min at room temperature. Afterwards, the cells were permeabilized with a blocking solution (PBS, 1% bovine serum albumin (BSA) (Sigma–Aldrich, Milan, Italy) and 0.2% Triton X-100 (ThermoFisher Scientific, Milan, Italy) for 20 min at room temperature, and then incubated overnight at 4 °C with mouse monoclonal anti-p50 subunit primary antibody (Santa Cruz Biotechnology, Italy) diluted at 1:100 in PBS, 1% BSA and 0.05% Triton X-100. Three washes in PBS of the coverslips were followed by 1 h incubation with goat anti-mouse IgG-Texas Red conjugated antibody (Southern Biotechnology, Italy) diluted at 1:100 in PBS, 1% BSA and 0.05% Triton X-100. Finally, the coverslips were washed three times in PBS and submitted to nuclear counterstain by 4,6-diamidino-2-phenylindole (DAPI), and then mounted with Vecta shield (Vector Labs). Fluorescence was examined under an AxioPlan (Zeiss, Oberkochen, Germany) light microscope equipped with epifluorescence at 200× and 400× magnification. The negative controls were obtained by omitting the primary antibody. Immunoreactivity of p50 was semi-quantified as the mean densitometric area of p50 signal into the nucleus and into the cytoplasm, by AxioVision 4.6 software measure program [89]. At least 100 synovial cells from each group were evaluated.

### 4.9. Statistical Analysis

Three independent experiments were carried out and the results were expressed as the mean ± SD of triplicate values for each experiment. Data normal distribution was evaluated by Shapiro–Wilk, D’Agostino and Pearson, and Kolmogorov–Smirnov tests.

Data from real-time PCR were evaluated by one-way ANOVA with a Tukey’s post-hoc test using 2^−ΔΔ*C*T^ values for each sample. Flow cytometry results were analyzed by ANOVA with Bonferroni post-hoc test.

All analyses were performed through the SAS System (SAS Institute Inc., Cary, NC, USA) and GraphPad Prism 6.1. A significant value was defined with a *p*-value < 0.05.

## 5. Conclusions

Growing evidence supports the relevance of synovitis in OA pathophysiology. Among the various factor involved in synovial membrane inflammation and in cartilage degradation during the development and the progression of OA, adipokines, miRNA, and oxidative stress play a crucial role. These findings induced us to deeper investigate the possible link between adipokines and some miRNA in oxidative stress regulation in human OA synovial cultures.

We firstly demonstrated the ability of visfatin and resistin to induce the gene expression of a pattern of pro-inflammatory cytokines (*IL-1β, IL-6, IL-17A* and *TNF-α*), MMPs (*MMP-1, MMP-13*), anti-oxidant enzymes (*SOD-2, CAT* and *NRF2*), as well as *miR-34a*, *miR-146a*, and *miR-181a.* Furthermore, they caused apoptosis and superoxide anion production, down-regulated the transcriptional levels of Col2a1 and the anti-apoptotic marker BCL2 and increased the p50 NF-κB activation.

Furthermore, we investigated the implication of *miR-34a, miR-146a*, and *miR-181a* as possible regulators of adipokines effects on the modulation of oxidative stress.

Finally, the use of NF-κB specific inhibitor points out the involvement of the pathway in adipokines-mediated effects.

In conclusion, altogether, these results confirm the role of visfatin and resistin in the induction of inflammation and cartilage degradation, and contribute to elucidate the existing crosstalk among adipokines, miRNA and oxidative stress.

However, further studies are required to deeper investigate this complex network and how this evidence can be useful to identify new possible therapeutic targets to reduce synovitis and cartilage degradation in OA.

## Figures and Tables

**Table 1:** Primers used for RT-qPCR.

**miRNA Genes**	**Cat. No. (Qiagen)**
*miR-34a*	MS00003318
*miR-146a*	MS00003535
*miR-181a*	MS00006692
*SNORD-25*	MS00014007
**Target Genes**	**Cat. No. (Qiagen)**
*IL-1β*	QT00021385
*IL-6*	QT00083720
*IL-17A*	QT00009233
*TNF-α*	QT00029162
*MMP-1*	QT00014581
*MMP-13*	QT00001764
*Col2a1*	QT00049518
*BCL2*	QT00000721
*SOD-2*	QT01008693
*CAT*	QT00079674
*NRF2*	QT00027384
*ACTB*	QT00095431

Abbreviations: miRNA = microRNA; SNORD-25 = Small Nucleolar RNA, C/D Box 25; IL-1β = interleukin 1β; IL-6 = interleukin 6; IL-17A = interleukin 17A; TNF-α = tumor necrosis factor-α; MMP-1 = matrix metalloproteinase 1; MMP-13 = matrix metalloproteinase 13; Col2a1 = type II collagen alpha 1 chain; BCL2 = B-cell lymphoma; SOD-2 = superoxide dismutase 2; CAT = catalase; NRF2 = nuclear factor erythroid 2 like 2; ACTB = actin beta.

## Supplementary Materials

Supplementary materials can be found at https://www.mdpi.com/1422-0067/20/20/5200/s1.
