# Supplementary material for: MicroRNA Mediate Visfatin and Resistin Induction of Oxidative Stress in Human Osteoarthritic Synovial Fibroblasts Via NF-κB Pathway
Source: Int J Mol Sci. 2019 Oct 20;20(20):5200. doi: 10.3390/ijms20205200 (PMC6829533; doi:10.3390/ijms20205200)
Supplement: Supplementary file 1 [file ijms-20-05200-s001.pdf]

Supplementary

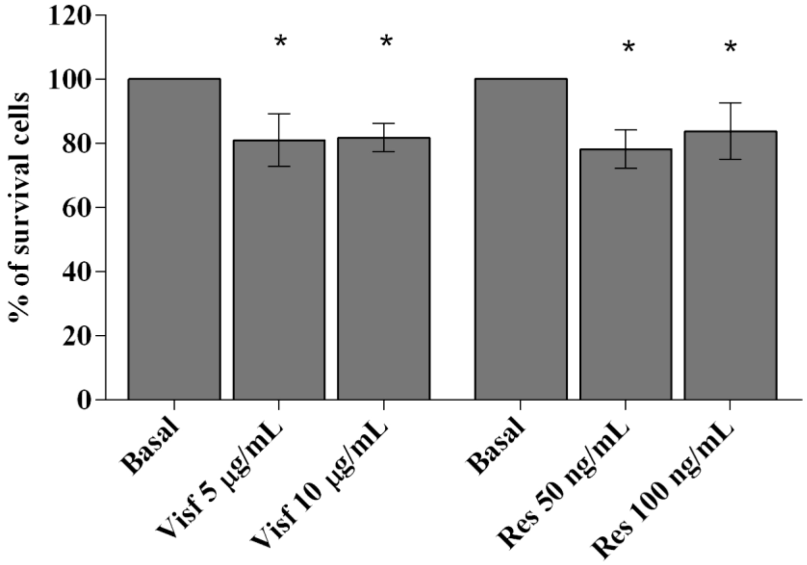

Figure S1. Evaluation of cell viability by MTT assay. Human osteoarthritic (OA) synovial fibroblasts were evaluated at basal condition and after incubation with visfatin (5 µg/mL and 10 µg/mL) and resistin (50 ng/mL and 100 ng/mL) for 24 h. The percentage was referenced to the ratio of the value of interest and the value of basal condition (Basal, cells without treatment), reported equal to 100. Data were expressed as mean ± standard deviation (SD) of triplicate values. *$p < 0.05$ versus basal condition. Visf = Visfatin, Res = Resistin.

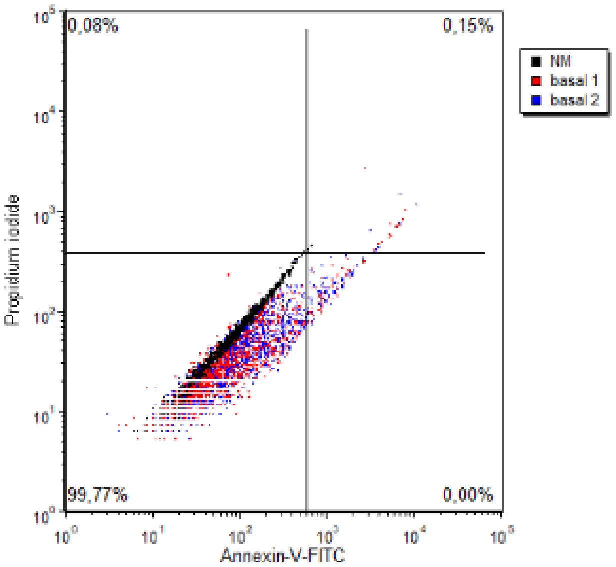

Figure S2. Apoptosis detection performed by the analysis at flow cytometry and measured with Annexin Alexa fluor 488 assay. A representative result showing the percentage of apoptotic, live and necrotic cells measured at basal condition following Annexin V and propidium iodide (PI) staining.

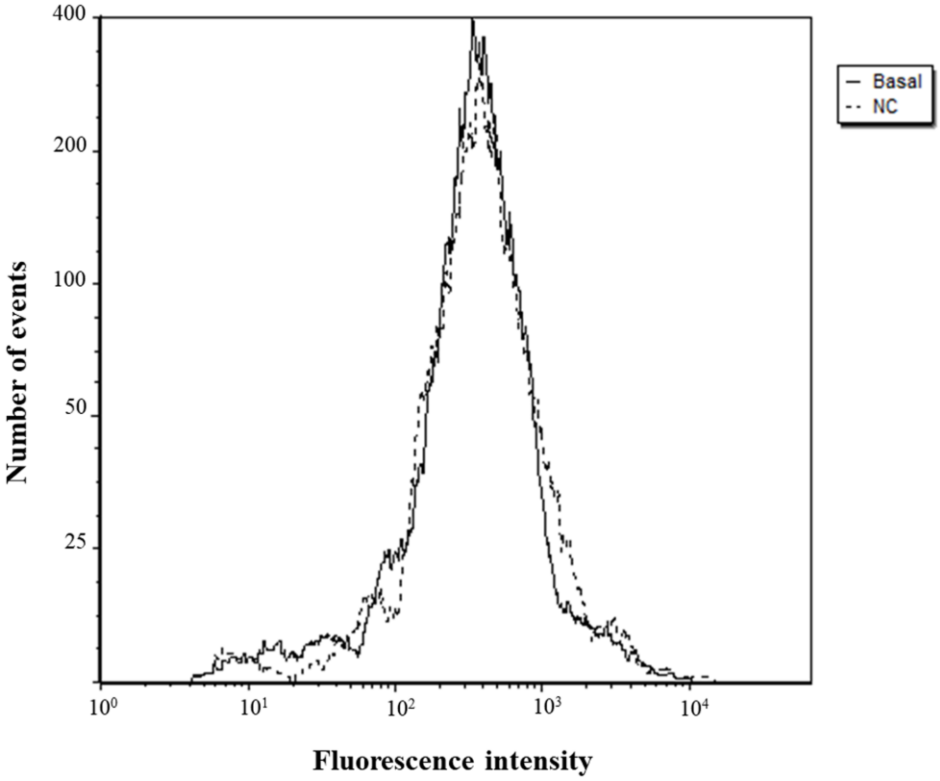

Figure S3. MitoSOX-based flow cytometric detection of mitochondrial ROS production in basal condition (Basal, cells without treatment) and negative control (NC).
